# Epigallocatechin-3-gallate inhibits osteoclastic differentiation by modulating mitophagy and mitochondrial functions

**DOI:** 10.1038/s41419-022-05343-1

**Published:** 2022-10-28

**Authors:** Jaganmay Sarkar, Manjusri Das, Md Sariful Islam Howlader, Prateeksha Prateeksha, Derek Barthels, Hiranmoy Das

**Affiliations:** Department of Pharmaceutical Sciences, Jerry H. Hodge School of Pharmacy, Texas Tech University Health Sciences Center, Amarillo, TX USA

**Keywords:** Mitophagy, Macroautophagy

## Abstract

A natural plant product, epigallocatechin-3-gallate (EGCG), was evaluated for its effectiveness in the regulation of osteoclastogenesis. We found that EGCG inhibited the osteoclast (OC) differentiation in vitro, and in primary bone marrow cells in a dose-dependent manner. Quantitative RT-PCR studies showed that the EGCG reduced the expression of OC differentiation markers. DCFDA, MitoSOX, and JC-1 staining revealed that the EGCG attenuated the reactive oxygen species (ROS), and mitochondrial membrane potential; and flux analysis corroborated the effect of EGCG. We further found that the EGCG inhibited mRNA and protein expressions of mitophagy-related molecules. We confirmed that the OC differentiation was inhibited by EGCG by modulating mitophagy through AKT and p38MAPK pathways. Furthermore, in silico analysis revealed that the binding of RANK and RANKL was blocked by EGCG. Overall, we defined the mechanisms of osteoclastogenesis during arthritis for developing a new therapy using a natural compound besides the existing therapeutics.

## Introduction

Osteoclasts (OC) are the multinucleated giant cells emerging from monocytic lineages and play a critical role in bone remodeling, resorption, and subsequently developing pathogenesis. In presence of MCSF, and RANKL, monocytes fuse together to differentiate into OC [1, 2]. Upon differentiation, the activated OC initiates the degradation of the bone matrix by secreting protons and lysosomal proteases like TRAP, MMP9, and Cathepsin K [2]. Hyperactivation of the OC disrupt in bone turnover and subsequently mediates destructive bone diseases like rheumatoid arthritis (RA), osteoporosis, bone cancers, and metastasis [3]. OC formation must be tightly regulated to inhibit the hyperactive bone resorption to control the initiation of bone diseases.

Autophagy, an intracellular process of self-degradation plays a critical role in maintaining intracellular homeostasis. The defective and damaged cellular components are recycled through the lysosomal machinery, and dysregulation of this may induce the pathogenesis of different diseases through this autophagic process [4–7]. In our previous studies, we have shown that during the OC differentiation autophagy-related molecules were increased [8]. Mitophagy is another important cellular process that maintains mitochondrial health and functions by eliminating superfluous mitochondria for the cell growth, and initiation of diseases [9, 10]. In numerous cancer cells, it was shown that upregulated autophagy and increased ATP levels require for their survival, activity, and function [5]. OC is the highly specialized cells that rapidly undergo apoptosis when terminally differentiated [11]. Mitochondria is producing ATP via oxidative phosphorylation and is the key source of energy for OC differentiation. Hence, OC markedly increases the mitochondrial functions during the remodeling to maintain bone homeostasis, and inhibition of autophagy causes apoptosis [12]. Thus, autophagy is an important mechanism in osteoclast differentiation and proves that autophagy and mitophagy are mutually co-related in the osteoclastogenesis process.

An imbalance in oxidant/antioxidant ratio produces oxidative stress, which induces the activation of several enzymes, and subsequent cellular responses through the production of reactive oxygen species (ROS), which also plays a diverse role in osteoporosis and arthritis [13, 14]. ROS also plays a critical role in bone resorption by inducing the differentiation of OC [15], whereas, scavenging of ROS prevents OC formation and also bone diseases [15], but the mechanisms remain unclear. Importantly, the activation of OC for bone resorption requires very high energy, to fulfill this requirement, a large number of mitochondria are needed in the OC for energy, and this process produces also ROS [14].

It was suggested that to reduce the side effect of hormonal therapy and some of pharmaceutical drugs, natural medicinal compounds might be useful for the treatment of patients with osteoporosis [16, 17]. Tea is extracted from the leaves of *Camellia sinensis* is one of the most popular beverages consumed throughout the world. More than 50-80% of tea polyphenol contain catechins such as (-)-epicatechin (EC), (-)-epicatechin-3-gallate (ECG), (-)-epigallocatechin (EGC), and (-)-epigallocatechin-3-gallate (EGCG) and have beneficial effects on the human health [18]. Among those, EGCG gained much attention because of its anti-oxidative, antiviral, anti-inflammatory, anti-aging, anti-cancer, neuroprotective effects, and has been efficacious for various human diseases [19–22]. It has been reported that regular tea drinkers have a reduced risk of hip fractures and have a higher bone mineral density (BMD) [23, 24]. However, the clear molecular mechanism and mode of action remain unclear, and herein, we report that EGCG inhibited the OC differentiation by regulating mitophagy and shed light on the mechanistic pathways.

## Results

### EGCG inhibits OC differentiation

Dose-dependent effect of EGCG on RAW264.7 cells viability was determined by MTT assay, and we did not notice any remarkable cell death with 50 µM concentration of EGCG after 24 h or 48 h of treatments (Fig. S1). When we performed the flow cytometry analysis using propidium iodide (PI) staining, no necrotic cells were found and the results resemble with MTT assays, and were shown in Fig. S13. We also confirmed that EGCG (50 µM) did not affect the apoptotic markers, cleaved caspase3, caspase9, and PARP compared to the control (Fig. S10).

**Fig. 1:**
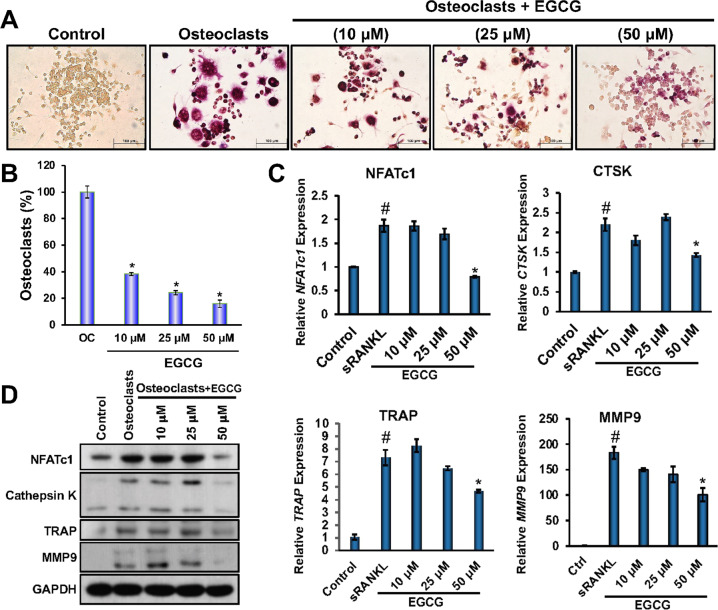
Dose-dependent inhibition of OC differentiation, along with its related molecules both in mRNA and protein levels by EGCG. **A** TRAP staining of OC differentiated cells in the presence or absence of EGCG. The dark purple color indicates the differentiated cells. **B** The percentage of TRAP-positive multinucleated OCs in each group is shown graphically. **C** Quantitative real-time PCR analysis for the expression of NFATc1, CTSK, TRAP, and MMP9 during OC differentiation. **D** Western blot for protein expression of NFATc1, cathepsin K, TRAP, and MMP9 molecules. For qRT-PCR β-actin, and for western blot GAPDH was used as a loading control. Results are mean ± SEM (*n* = 3). (#), *p* < 0.05 compared with basal condition; and (*), *p* < 0.05 compared with induced OC differentiated condition.

Multinucleated cells were visible after differentiation of OC, which is the hallmark of differentiation, we found that the differentiated cells were multinucleated, and those cells were positive for TRAP (Fig. 1A). To determine the effect of EGCG on the OC differentiation, RAW264.7 cells were treated with EGCG at various concentrations (10 µM, 25 µM, and 50 µM) for 6 days in an OC differentiation medium, and followed by the TRAP staining. We found that the EGCG significantly inhibited the number of TRAP-positive OC cells in a dose-dependent manner (Fig. 1A, B).

Separate sets of experiments (for qRT-PCR and WB) were performed to determine the effect of EGCG on the mRNA and protein expression of OC-related markers like NFATc1, Cathepsin K, TRAP, and MMP9. The results obtained from the qRT-PCR, and western blot studies showed that EGCG significantly inhibited both the mRNA and the protein expressions of the OC-related markers (Fig. 1C, D). The densitometric analysis for WB were shown in Fig. S2. Based on the above results we found that 50 µM EGCG is optimum for inhibiting the OC formation and further experiments were performed using this concentration.

To investigate the effect of EGCG on inflammation, we have performed the qRT-PCR for the various relevant pro-inflammatory and anti-inflammatory factors in the presence or absence of EGCG (50 μM) after the osteoclastic (OC) differentiation. We found that the EGCG downregulated the pro-inflammatory markers such as COX-2, IL-1β, IL6, MACP1, P65, and TNF-α, and upregulated the anti-inflammatory markers such as Arg1, IL-4R, IL-10, and Ym1 during the OC differentiation. The qRT-PCR data is shown in Fig. S11., and Fig. S12.

### EGCG inhibited intracellular and mitochondrial ROS formation during OC differentiation

**Fig. 2:**
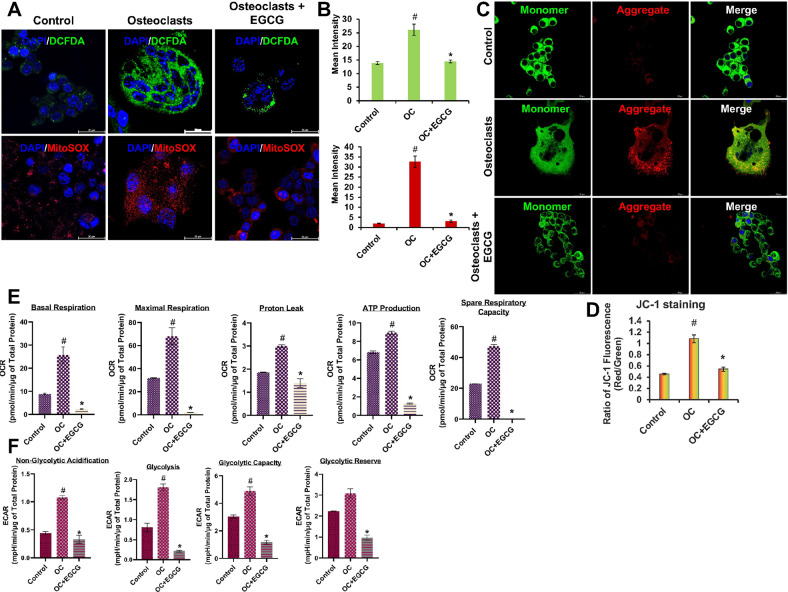
EGCG inhibits ROS production, mitochondrial membrane potential and improves the mitochondrial functional activity during OC differentiation. **A** ROS generation was detected by DCFDA and mitoSOX staining. **B** Quantification graph for mean fluorescence intensity (green for DCFDA and red for mitoSOX staining). **C** JC-1 staining for mitochondrial membrane potential measurement. **D** Quantification of membrane potential by measuring the ratio of red and green fluorescence intensity graph. Seahorse flux analysis results are shown graphically. **E** Levels of oxygen consumption rate (OCR), which is an indicator of mitochondrial respiration at basal, maximal respiration, proton leak, ATP production, and spare respiratory capacity conditions. **F** Levels of extracellular acidification rate (ECAR) in the non-glycolytic acidification, glycolysis, glycolytic capacity, and glycolytic reserve. Results are shown in mean ± SEM (*n* = 3). (#), *p* < 0.05 compared with basal condition; and (*), *p* < 0.05 compared with induced OC differentiated condition.

Next, we evaluate the effect of EGCG on intracellular and mitochondrial ROS formation during OC differentiation. The RAW264.7 cells were treated with 50 µM of EGCG in presence of differentiation media for 6 days, and after differentiation, cells were stained with DCFDA and mitoSOX. Results obtained from DCFDA and mitoSOX staining showed that during the OC differentiation intracellular and mitochondrial ROS formation was increased, and those parameters were significantly reduced after the addition of EGCG to the culture (Fig. 2A, B).

### Effect of EGCG on mitochondrial dysfunction during OC differentiation

Mitochondria play an essential role in the survivability of cells, including the production of ATP, and the OC differentiation is the high energy (ATP) dependent process [25]. To determine the effect of EGCG on the mitochondrial membrane potential (MMP) during the OC differentiation, JC-1 staining was performed. Our results showed that during OC differentiation the MMP was increased, which was significantly reduced after treatment with EGCG (Fig. 2C, D).

### Effect of EGCG on oxygen consumption rate and extracellular acidification rate during OC differentiation

We performed the Seahorse analysis to determine the glycolysis and oxidative phosphorylation (through oxygen consumption) simultaneously in live cells during the course of OC differentiation. Our results revealed that during OC differentiation the oxygen consumption rate (OCR) was increased and after the addition of EGCG, OCR parameters were significantly decreased, which were represented by mitochondrial respiration at basal respiration, maximal respiration, proton leak, ATP production, and spare respiratory capacity (Fig. 2E, Fig. S3A).

In separate sets of experiments, we also determined the effect of EGCG on the glycolytic stress during the OC differentiation by using Seahorse XF Glycolysis Stress Test Kit. Data obtained from our experiments confirmed that mitochondrial metabolic functions were also affected during the OC differentiation, and EGCG played a key role in this process, reflected by the increased levels of extracellular acidification rate (ECAR) in the non-glycolytic acidification, glycolysis, glycolytic capacity, and glycolytic reserve during OC differentiation, which were significantly decreased after addition of EGCG (Fig. 2F, Fig. S3B).

### Effect of EGCG on the regulation of autophagy during OC differentiation

**Fig. 3:**
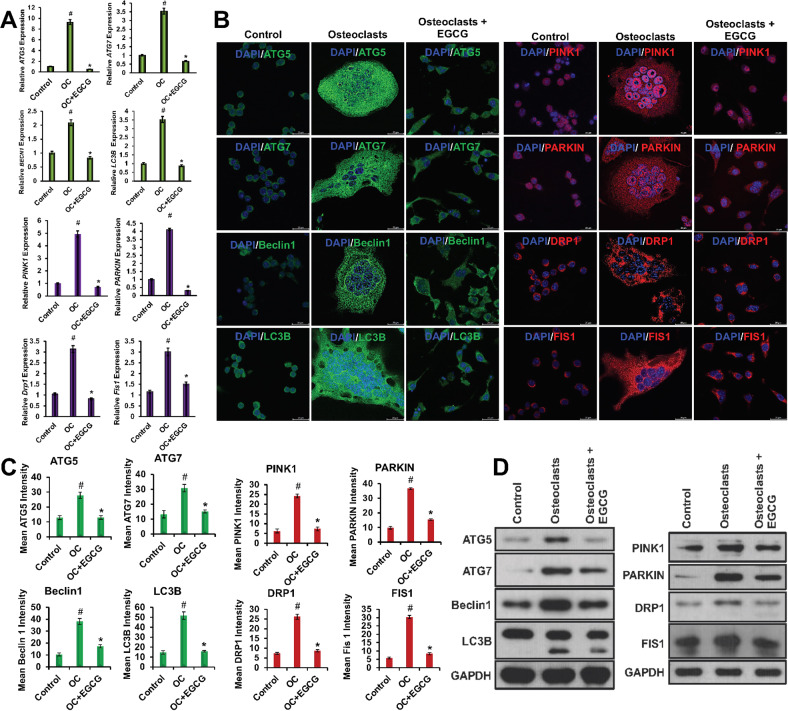
EGCG inhibits autophagy and mitophagy-related molecules. **A** qRT-PCR for various gene expressions is presented graphically. **B** Representative IF staining images is presented. **C** Quantified mean intensity graphs for IF staining is presented graphically. **D** WB images are shown for protein levels of ATG5, ATG7, Beclin1, LC3B, PINK1, PARKIN, DRP1, and FIS1 during the OC differentiation in the presence or absence of EGCG. For qRT-PCR, β-actin, and WB, GAPDH was used as a loading control. Results are shown in mean ± SEM (*n* = 3). (#), *p* < 0.05 compared with basal condition; and (*), *p* < 0.05 compared with induced OC differentiated condition.

Autophagy acts as a critical regulator during OC differentiation [8, 25]. Here, we determined the effect of EGCG on the expression of some important autophagic molecules such as ATG5, ATG7, Beclin1, and LC3B during the OC differentiation using a quantitative RT-PCR method. Our results showed that during the OC differentiation the mRNA expressions of ATG5, ATG7, Beclin1, and LC3B were increased several folds, and upon the addition of EGCG the autophagy markers were significantly decreased (Fig. 3A).

In separate sets of experiments, we also determined the effect of EGCG by using immunofluorescence studies. We found that the addition of EGCG significantly inhibited the mean fluorescence intensity of the ATG5, ATG7, Beclin1, and LC3B expressions with respect to the OC differentiation alone (Fig. 3B, C).

In other sets of experiments, we performed the WB analyses to determine the effect of EGCG on the protein levels of the above-mentioned autophagic molecules. These data also corroborated with the previous results that were obtained from the qRT-PCR and IF studies (Fig. 3D). The densitometric analyses of WB were shown in Fig. S4A.

### Effect of EGCG on the regulation of mitophagy during OC differentiation

OC undergoes apoptosis when terminally differentiated, and requires high energies, possesses copious mitochondria [11]. Mitophagy is the process that maintains mitochondrial health and functions by the elimination of superfluous mitochondria [9, 10]. We determined the expressions of PINK1 and PARKIN that were associated with mitophagy, and also determined the levels of DRP1, and FIS1 expressions that were associated with mitochondrial fission, and are responsible for the mitochondrial fragmentation during the OC differentiation. First, we performed qRT-PCR studies to determine the effect of EGCG on the mRNA expressions of PINK1, PARKIN, DRP1, and FIS1. Our results showed that the mRNA expressions of PINK1, PARKIN, DRP1, and FIS1 were increased during the OC differentiation, and upon the addition of EGCG those mitophagy-related molecular expressions were significantly decreased (Fig. 3A).

We next determined the effect of EGCG on mean fluorescence intensities of PINK1, PARKIN, DRP1, and FIS1 using IF studies. We found that EGCG inhibited the mean fluorescence intensity of the PINK1, PARKIN, DRP1, and FIS1 expressions compared to the OC differentiation alone (Fig. 3B, C).

Next, in separate sets of experiments, we determined the effect of EGCG on PINK1, PARKIN, DRP1, and FIS1 molecules at the protein level, and our results corroborated with the results of qRT-PCR and IF studies (Fig. 3D). The densitometric analysis of western blots were shown in Fig. S4B.

### Effect of EGCG on the trafficking of PINK1, PARKIN, DRP1, and FIS1 during OC differentiation

**Fig. 4:**
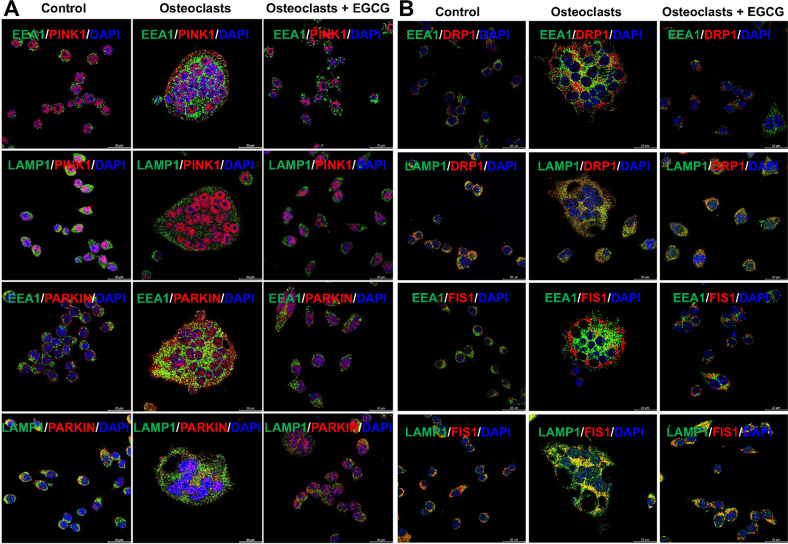
Effect of EGCG in the intracellular trafficking of mitophagy- and mitochondrial fission-related molecules. **A** Representative co-immunostaining images are presented for PINK1 and PARKIN with EEA1 or LAMP1. **B** Representative co-immunostaining images are presented for DRP1 and FIS1 with EEA1 or LAMP1.

We next determined the effect of EGCG in the intracellular trafficking of mitophagy molecules such as PINK1, PARKIN, DRP1, and FIS1 in early endosomes with EEA1 (specific marker for early endosome), and late endosomes/lysosomes using LAMP1 (specific marker for late endosome/lysosome) respectively. Our results indicated that in control cells there was no colocalization of PARKIN with the EEA1 molecule. After the OC differentiation PARKIN co-localized with EEA1, and remained in the early endosome, however, when cells were differentiated in presence of EGCG the colocalization of PARKIN was not in the early endosome, they were partially trafficked to the late endosome/lysosomes, which were partially colocalized with the LAMP1, however, we didn’t find any remarkable co-staining in PINK1 trafficking using either of the early or late endosomal markers, maybe the level was very low (Fig. 4A).

We further verified the colocalization of mitochondrial fission-related markers such as DRP1 and FIS1 using EEA1 and LAMP1. We found that the DRP1 was not co-localized with the EEA1 in control cells, however, after OC differentiation DRP1 was distributed both in the early and late endosomes. The colocalization amount in early endosomes was relatively lower compared to the non-colocalization with EEA1. However, after the addition of EGCG, we found that the colocalization was similar to the control cells DRP1 was not trafficked to the early endosome and did not colocalize with the EEA1 (Fig. 4B). When co-stained DRP1 with LAMP1, it showed a clear colocalization in control cells, and in OC differentiated cells in the presence or absence of EGCG (Fig. 4B). Trafficking distributions were observed with FIS1 were similar to the distributions of DRP1without or with differentiation, in the presence or absence of EGCG (Fig. 4B).

### Effect of EGCG on the primary BM cells isolated from mice

**Fig. 5:**
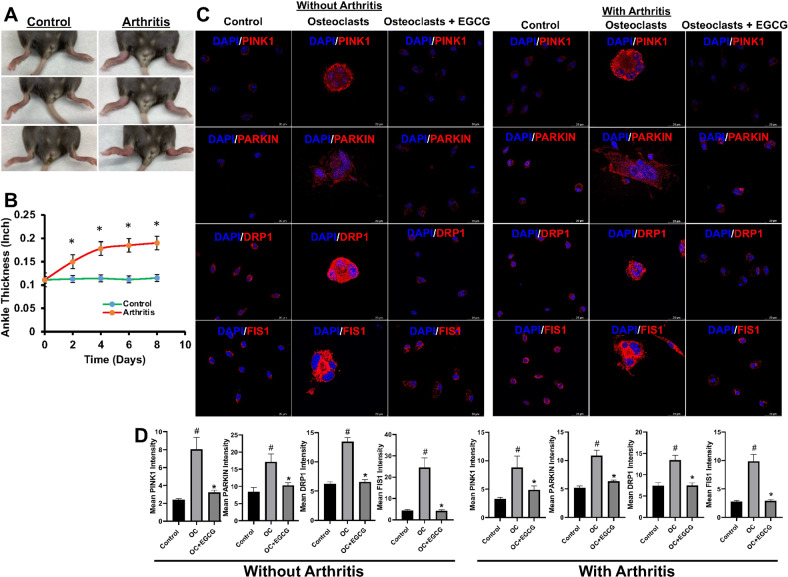
Effect of EGCG in the expression of mitophagy-related molecules in primary BM isolated from without or with K/BxN serum-induced arthritis mice. **A** Representative images of the K/BxN serum-induced arthritic inflammation of limbs (in C57BL/6 female mice). **B** Measured ankle thickness during the course of arthritis development is shown graphically. **C** Representative IF staining images of PINK1, PARKIN, DRP1, and FIS1 were shown during the OC differentiation of with or without arthritis mice in the presence or absence of EGCG. **D** Quantified mean intensity graph for IF staining shown. Results are shown in mean ± SEM (*n* = 3). (#), *p* < 0.05 compared with basal condition; and (*), *p* < 0.05 compared with induced OC differentiated condition.

To validate our in vitro data, K/BxN serum-induced arthritis in mice (C57BL/6) was generated and compared with the littermate control mice (Fig. 5A, B). The BM cells from all mice were isolated, cultured, and differentiated to OC using the differentiation media in the presence or absence of EGCG on coverslips inserted into wells of a 6-well plate. After differentiation, some of the coverslips were fixed, and the IF studies were performed for PINK1, PARKIN, DRP1, and FIS1, and the rest of the coverslips were used for live-cell imaging (without any fixation) to determine the ROS production and MMP levels by staining with DCFDA, mitoSOX, and JC-1, respectively.

The results obtained from DCFDA, and mitoSOX staining showed that the addition of EGCG inhibited the intracellular and mitochondrial ROS formation in the primary BM cells derived from both the control and K/BxN serum-induced arthritis mice (Fig. S5A–D). Similarly, JC-1 staining also showed that EGCG treatment significantly inhibited the MMP level (Fig. S6A–D).

In separate sets of experiments, we also determined the changes in the mitophagy markers using the primary BM cells, and our results showed that the expressions of PINK1, PARKIN1, DRP1, and FIS1 were increased during the OC differentiation with respect to the untreated conditions, and the enhanced expressions of these molecules were reduced upon addition of the EGCG to the culture (Fig. 5C, D). This result corroborates our in vitro results however, the level of differentiation (number of multinucleated cells and number of nuclei per differentiated cells) was much lower in primary cells compared to the in vitro cells.

### Effect of EGCG on the signaling pathways related to OC differentiation

**Fig. 6:**
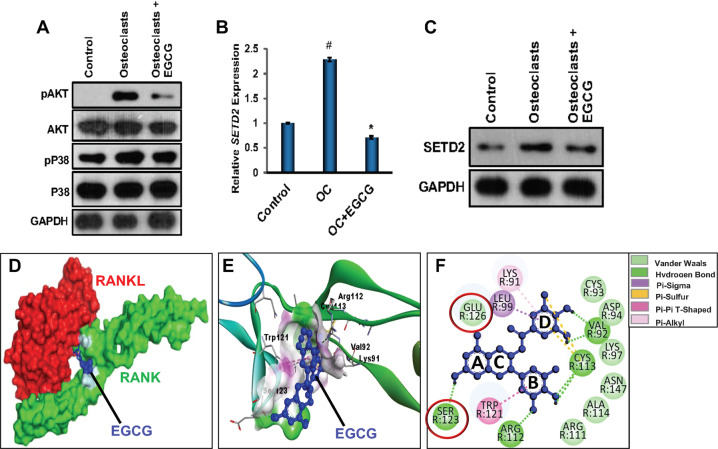
Potential mechanisms, and in silico analyses for the mode of action of EGCG in the RANKL/RANK induced OC differentiation. **A** Western blot images are shown to evaluate the effect of EGCG in the AKT and p38MAPK signaling pathways. **B** qRT-PCR for determining the effect of EGCG on the expression of the SETD2 gene. **C** Western blot image showing SETD2 protein expression level. For qRT-PCRβ-actin, and for WB, GAPDH was used as a loading control. Results are shown in mean ± SEM (*n* = 3). (#), *p* < 0.05 compared with basal condition; and (*), *p* < 0.05 compared with induced OC differentiated condition. **D** 3D surface representation of ECGC-RANK-RANKL complex. **E** 3D ribbon representation of ECGC-RANK complex. **F** 2D representation of EGCG-RANK complex. Blue, red, and green color represent EGCG, RANKL, and RANK, respectively.

During the analysis of signaling pathways, we found that the activation of AKT and p38MAPK pathways were the major pathways involved in OC differentiation. We have determined the effect of EGCG on those pathways. Our results showed that during osteoclastogenesis the phosphorylation of AKT and p38MAPK were remarkably increased, and those activations were inhibited upon the addition of EGCG, however, EGCG did not show any discernable changes in the levels of total proteins (Fig. 6A). In our previous work, we showed an increased level of SETD2, an epigenetic regulator is involved in the regulation of OC differentiation [26]. Therefore, in this study, we also determined the effect of EGCG on the mRNA expression and protein level of SETD2 and found that the EGCG inhibited both the mRNA expression and protein level of the SETD2 molecule (Fig. 6B, C). The densitometric results for the western blots were shown in Fig. S7.

### Effect of EGCG in binding with RANK and RANKL

To define the binding of EGCG with the RANK, molecular docking analysis was performed using in silico analysis methods. The in silico analysis results demonstrated that the EGCG is exactly bound with the extracellular domain (residues 30-197) of RANK in the CRD (cysteine-rich domain) 2 and CRD3 region (Fig. 6D–F), which are particularly involved in RANKL interaction. EGCG is firmly gripped into the pocket of RANK with Gibbs free energy of −5.57 kcal/mol, and an inhibition constant of 88.73 µM. The interaction energy profiles were summarized in Fig. S8A, B. It was observed that the EGCG mainly blocked RANKL interaction with RANK by establishing the hydrogen bond with Ser123 amino acid residue and Van der Waal interaction with Glu126 of RANK, as these amino acids usually interacted with Lys180 of RANKL. Interestingly, EGCG also formed six hydrogen bonds, seven Van der Waal interactions, two sulfur bonds, and some other pi-pi interactions with RANK. The amino acid residues of RANK involved in specific interactions were summarized in Fig. S8C, D. A higher number of hydrogen bonds revealed a strong binding affinity of EGCG to RANK. So, EGCG might compete with RANKL under physiological conditions to block RANK-RANKL signaling. With this blockade, EGCG inhibited the OC differentiation process of myeloid cells.

## Discussion

Excessive OC differentiation causes an imbalance in bone resorption and that helps in the development of bone-related diseases like arthritis and osteoporosis. Well-known hormonal and pharmaceutical drugs such as estrogen, calcitonin, bisphosphonates, denosumab, etc., which are commonly used for the treatment of patients with those diseases have serious side effects and toxicity [27]. Recently, several functional foods, natural medicinal plant extract, and their derived compounds such as polyphenols, alkaloids, flavonoids, and polysaccharides were the largest targets, and gain focus to develop new therapies for several diseases because of their anti-inflammatory, antiproliferative, antiangiogenic, antioxidant, and specially bioavailability and nontoxic properties [28–31]. Our goal is to verify one of those natural products whether has the potential to minimize the differentiation of osteoclasts and if so, to find out what are the mechanisms by which that compound mediates the functions to develop an effective future therapeutic for those pathological conditions.

EGCG, tea catechin is well-known for its various biological functions and is beneficial for human health including bone physiology [19–22, 32, 33]. Previous studies have shown that EGCG plays an important role in the death of OC [34, 35], however, the molecular mechanism is not yet clear. Therefore, in this study, we determined the effect of EGCG in the OC differentiation using RAW264.7 cells, and primary BM cells collected from normal (C57BL/6 background) and K/BxN serum-induced arthritis in mice respectively. Our TRAP staining results showed that EGCG inhibits the OC differentiation in a concentration-dependent manner (Fig. 1A, B). The activated OC induces the degradation of bone matrix by secreting protons, and lysosomal proteases like the TRAP, MMP9, and Cathepsin K [36]. Whereas NFATc1, a transcription factor is sturdily expressed during the RANK/RANKL-induced OC differentiation [37]. To confirm the results obtained from TRAP staining, and the previous findings, we determined the effect of EGCG on the expression of molecules such as NFATc1, TRAP, MMP9, and Cathepsin K. Our results showed that EGCG inhibits significantly both protein and mRNA expression of the NFATc1, Cathepsin K, MMP9, and TRAP molecules (Fig. 1C, D).

Evidence supports that the mitochondria play a key role in the intracellular signaling for the regulation of immune response, as well as maintaining the OC differentiation and maturation [25, 38]. Increased mitochondrial activation and ATP supply are essential for OC differentiation. Mitochondria is the major source of ATP production, it also produces ROS as a by-product, which is harmful to the cells [39]. Previous findings revealed that ROS production generates oxidative stress, promotes OC differentiation [40], and damages mitochondrial function [39]. In this study we determined the effect of EGCG in the following aspects; (i) intracellular and mitochondrial ROS production; (ii) mitochondrial membrane potential; and (iii) mitochondrial function during osteoclastogenesis, which are crucial for bone remodeling and maintaining homeostasis. To determine the effect of EGCG on the intracellular and mitochondrial ROS production, we performed the DCFDA and mitoSOX staining in cell line and primary BM cells, and our results showed that the EGCG inhibited the intracellular and mitochondrial ROS production in both cells, and EGCG acts as an antioxidant during the OC differentiation (Fig. 2A, B; and Fig. S5A–D). Next, we determined the effect of EGCG on the MMP during the OC differentiation, and our results showed that MMP increased during the OC differentiation, which was reduced upon treatment with EGCG (Fig. 2C, D; and Fig. S6A–D). In addition, we also performed the Seahorse flux analysis to determine the effect of EGCG on the mitochondrial glycolytic and non-glycolytic stress, and also the ATP production. Our result showed that during OC differentiation ATP production was increased, which was controlled by the EGCG and also improved the overall mitochondrial functions (Fig. 2E, F).

Autophagy is an intracellular self-degradation process, which breaks and recycles the intracellular organelles and proteins, plays a critical role in the maintenance of intracellular metabolism and homeostasis, through the lysosomal machinery, and dysregulation of this process induces the pathogenesis of various diseases like cancer, infections, heart, and neurodegeneration [4–7]. Our previous studies showed that autophagy was enhanced, and autophagic activity positively correlated with OC differentiation [8]. In this present study, we have shown the expression of some autophagic molecules such as ATG5, ATG7, Beclin1, and LC3B during OC differentiation by qRT-PCR, IF, and WB. Our results revealed that during the OC differentiation the mRNA and protein expression of the autophagic molecules were increased, which was decreased with the EGCG. This result confirmed that EGCG inhibited OC formation by inhibiting autophagy (Fig. 3A–D).

The relationship between ROS-mediated signaling and autophagy is very complex, and depends on the cell type and disease conditions. A previous study has shown that the mitochondrial function during OC differentiation was controlled by autophagy [25]. Mitophagy, a specialized form of autophagy, is another important cellular mechanism that selectively crumbles the copious mitochondria and helps mitochondrial metabolism to maintain mitochondrial health and functions [9, 10]. Small GTPase gathers in the outer mitochondrial membrane and induces mitophagy to supply ATP from the mitochondria upon increased oxidative phosphorylation [41]. Increased oxidative phosphorylation in the mitochondria generates enormous amounts of ROS and induces mitochondrial damage that critically controls the OC differentiation process. In addition to increased ATP production, autophagy also maintains mitochondrial function via mitophagy. In mammalian cells, the most studied mitophagy-associated proteins are PTEN-induced putative kinase 1 (PINK1), and PARKIN [42]. In mitochondrial dysfunction, PINK1 (a serine/threonine kinase), helps in the recruitment of the PARKIN (an E3-ubiquitin ligase), and controls the PARKIN-mediated mitophagy to promote their elimination [43]. A study on Parkinson’s disease showed that mutation in the PINK1/PARKIN triggered mitochondrial dysfunctions, and led to nigral neurodegeneration to promote the early commencement of the disease [44]. It was also reported that the downregulation of PARKIN was associated with osteogenic differentiation of adipose-derived mesenchymal stem cells (MSCs) and affected the expression of bone morphogenetic protein 2 and collagen 1 [45]. These findings encouraged us to determine the changes in the expression of PINK1/PARKIN during the OC differentiation, and we performed the qRT-PCR, IF, and WB. We found that the PINK1 and PARKIN expression was increased during the OC differentiation, which was minimized upon the addition of EGCG (Fig. 3A–D; and 5C, D). Mitochondrial mass and mitochondrial homeostasis are regulated by the fusion and fission processes. Mitochondrial integration depends on fusion, whereas fission enables the fragmentation of mitochondria. The Dynamin family of GTPases, particularly DRP1 is involved in controlling mitochondrial fission and promoting the fragmentation of mitochondria, which is very important for several pathophysiological conditions [46]. Fission protein 1 (FIS1), a mitochondrial fission protein is an important component for the regulation of apoptotic, and mitophagy pathways [47]. Previous reports showed that the DRP1/FIS1 interaction is essential for mitochondrial fission [48, 49]. In this study, we showed that the DRP1 and FIS1 expressions were increased during OC differentiation, which was reduced after the addition of EGCG (Fig. 3A–D; and 5C, D).

It is already known that RANK binds to the RANKL to initiate the myriad of downstream signaling pathways and regulates the osteoclastogenesis process. In this study, we also determined the effect of EGCG on the signaling molecules associated with OC differentiation. Our results showed that the phosphorylation of AKT and p38MAPK significantly increased during the OC differentiation, but upon addition of EGCG inhibited the phosphorylation of AKT and p38MAPK without any remarkable changes in the total amounts (Fig. 6A). In our previous research, we showed that the SETD2, an epigenetic regulator is upregulated during OC differentiation and after induction of arthritis and SETD2’s regulatory mechanisms [26]. In this study, we found that the EGCG inhibited the mRNA and protein expressions of SETD2 (Fig. 6B, C). Next, we analyzed the mode of binding of EGCG, and/or the role of EGCG in the binding with RANK/RANKL, through in silico molecular docking analyses, and we found that the EGCG binds with the RANK in the same position where RANKL binds. More precisely, EGCG exactly binds with the extracellular domain (residues 30-197) of RANK in the CRD2 and CRD3 regions, which are particularly involved in RANKL interaction (Fig. 6D–F), and EGCG competes with RANKL under the physiological condition to block RANK-RANKL signaling.

## Conclusion

In sum, we showed that the EGCG inhibited the OC differentiation by reducing the intracellular and mitochondrial ROS production, alteration in the autophagy-dependent mitochondrial functions, mitochondrial membrane potential, and mitophagy-related molecular expressions. Additionally, we showed that the EGCG inhibited the AKT, p38MAPK signaling pathways, and inhibited the epigenetic regulator, SETD2 molecule. We also showed how the EGCG inhibited the RANK and RANKL binding, which is essential for OC differentiation. Overall, these findings will help in the development of a new therapeutic target in near future for treating destructive bone diseases.

## Materials and methods

### Reagents and antibodies

RAW264.7 cell line was obtained from American Type Culture Collection (ATCC, #TIB-71). Soluble (s) RANKL (#315–11) and MCSF (#315–02) were obtained from Pepro Tech Incorporation. (-)-Epigallocatechin gallate (#ab120716), antibodies of Cathepsin K (#ab188604), DRP1 (#ab184247), MMP9 (#ab38898) and ATG5 (#ab12994) were purchased from Abcam. BCA protein assay kit (#23225), propidium iodide (# p3566), JC-1 Dye (#T3168), mitoSOX red compound (#M36008), TRAP (#PA5-116970) antibody were the product of Thermo Fischer Scientific. 4% paraformaldehyde (PFA, #sc-281692) was the product of Santa Cruz Biotechnology. The tartrate-resistant acid phosphatase (TRAP) Assay Kit (#387A-1KT), Triton X-100 (#T8787), RIPA lysis buffer (#20-188), 2′,7′-dichlorodihydrofluorescein diacetate (DCFDA, #4091-99-0) were obtained from Sigma-Aldrich. 4,6-diamidino-2-phenylindole, dihydrochloride (DAPI, #D1306), TRIzol reagent (#15596026) were purchased from Invitrogen Corporation. Antibodies for Beclin1 (#4122 S), ATG7(# 8558 S), phospho AKT (#4060 S), AKT (#4691 S), phospho p38MAPK (#4511 S), p38MAPK (#9212 S), LC3B (#12741 S), PINK1 (#6946 S), PARKIN (#2132 S), NFATc1 (#4389 S), SETD2 (#23486) and GAPDH (#2118 S), cleaved caspase3 (#9661 S), caspase9 (#9502 S), PARP (#9542 S), HRP (horseradish peroxidase)-labeled secondary antibodies (#7074, and #7076) were purchased from Cell Signaling Technology. PVDF membrane (Bio-Rad Incorporation, #1620115). Enhanced chemiluminescence (ECL, #RPN2232) Amersham Pharmacia Biotechnology. High-Capacity RNA-to-cDNA Kit (#4387406), SYBR Green PCR Kit (#4309155), FIS1 (#10956-1-AP) antibody were the product of Applied Biosystems.

### Cell culture and osteoclast differentiation

RAW264.7 cells were grown in culture media (DMEM supplemented with 10% fetal bovine serum and 1% penicillin-streptomycin) at a 37 °C incubators containing 5% CO_2_ and sub-cultured according to experimental requirements. To perform the osteoclastic differentiation, RAW264.7 cells were seeded on the coverslip at a density of 1 × 10^4^ cells per well in a six-well plate and then treated with sRANKL (40 ng/ml) containing complete media for 6 days. The culture media (in the presence or absence of stimuli and/or inhibitors) was replaced by fresh media every two days.

### Cell viability assay

The cell viability was measured by MTT assay to determine the effect of EGCG. In short RAW264.7 cells (2 × 10^3^/well) were seeded in a 96-well plate and incubated for 24 h at 37 °C with 5% CO_2_ incubator. Next day, the media were replaced with or without varying concentrations (5, 10, 25, 50, and 100 µM) of EGCG, and incubated for 24 h and 48 h. Then, cell viability was analyzed via MTT assay using a Roche Cell Proliferation Kit I (Roche Diagnostics, Basel, Switzerland, #11465007001) following the manufacturer’s protocol. The absorbance was measured using a microplate reader (Synergy 2, BioTeK Instruments Inc, Winooski, USA). The results were expressed as the percentage viability according to the following formula: % Viability = 100 × (absorbance of treatment/absorbance of control). All the experiments were performed in triplicate.

### Flow cytometry

Flow cytometry was carried out to further investigate the effect of EGCG on the viability of RAW264.7 cells using the propidium iodide (PI) staining. In short, RAW264.7 cells (1 × 10^5^/well) were seeded in a 6-well plate and incubated for 24 h at 37 °C with 5% CO_2_ incubator. Next day, the media were replaced with or without varying concentrations (50, and 100 µM) of EGCG, 400 µM of hydrogen peroxide (H_2_O_2,_ a positive control), and incubated for 24 h. The cells were harvested and washed three times with 1× PBS followed by staining with PI (10 µg/ml) for 15 min. After that, the cells were washed and analyzed by using a FACSVerse flow cytometer (BD Biosciences). At least 10,000 events were acquired for each sample for analysis using BD FACSuite software (BD Biosciences).

### TRAP staining

Osteoclastic differentiation was determined by TRAP staining according to the manufacturer’s protocol. Briefly, RAW264.7 cells (1 × 10^4^ cells/well), were cultured on a coverslip in a 6-well plate for differentiation to osteoclasts using OC differentiation media in the presence or absence of EGCG. On the 6th day of differentiation, coverslips were washed with 1× PBS and fixed with 4% PFA for 20 min at room temperature, and then, staining was performed using a TRAP assay kit following the manufacturer’s protocol. In short, the preparation of the incubation solution was made by mixing sodium nitrite solution, Fast Garnet GBC base solution, acetate solution, naphthol AS-BI phosphate solution, and tartrate solution in 37 °C pre-warmed deionized water. This incubation solution was added to the coverslips and incubate for 1 h at 37 °C protecting them from light. Finally, the coverslips were thoroughly washed with water, mounted on a glass slide, and examined under a light microscope, (Olympus Corporation of the Americas, Waltham, MA, ix81). TRAP-positive cells (purple) containing at least three nuclei were considered differentiated OC. Three separate sets of experiments were performed.

### Protein isolation and western blot analysis

RAW264.7 cells (1 × 10^5^ cells/well) were grown in a 6-well plate for the desired time and with treatment conditions, and cell lysates were collected for western blot (WB) using RIPA buffer. Briefly, cells were washed three times with ice-cold 1× PBS and lysed using the pre-cooled RIPA lysis buffer on ice for protein extraction. Pellets were removed after centrifugation. The protein concentrations were measured using the BCA protein assay kit, according to the manufacturer’s protocol. Equal amounts of proteins (40 μg) were separated by SDS-PAGE gel electrophoresis and transferred to the PVDF membrane. Then membranes were blocked with 5% non-fat milk for 1 h at room temperature and incubated with primary antibodies overnight at 4 °C. After that, membranes were incubated with appropriate HRP-conjugated secondary antibodies for 2 h at room temperature. Protein bands were visualized by ECL. Three separate sets of experiments were performed and densitometric analysis was performed by using the Fiji Image J software.

### RNA extraction and real-time PCR

RAW264.7 cells (1 × 10^5^ cells/well) were grown in a six-well plate in OC differentiation media in the presence or absence of desired concentration of EGCG. Total RNA was extracted by TRIzol reagent by following the manufacturer’s protocol. A total 1 µg of RNA was used for the synthesis of cDNA using a high-capacity RNA-to-cDNA Kit according to the manufacturer’s protocols. Real-time PCR amplification reactions were performed using the SYBR Green PCR Kit. The relative mRNA expression of each target gene was quantified by calculating Ct (threshold cycle) values and normalized by β-actin levels. Each sample was analyzed in triplicate. The primer pairs were purchased from Integrated DNA Technologies (IDT). The primer sequences were provided in Fig. S9.

### Immunofluorescence staining

To determine the expressions of the autophagy- and mitophagy-related molecules, and their colocalization was determined by co-staining with early endosomal (EEA1), and late endosomal (LAMP1) markers after OC differentiation in the presence or absence of EGCG using immunofluorescence (IF) staining. In brief, RAW264.7 and primary BM cells (1 × 10^4^ cells/well) isolated from C57BL/6 mice were cultured on sterile coverslips inserted into a well of a 6-well plate using OC differentiation media in presence or absence of EGCG. On day 6 of differentiation, cells were fixed with 4% PFA for 30 min. After washing with 1× PBS, cells were permeabilized with 0.1% Triton X-100 for 15 min at room temperature and blocked with 1% BSA for 30 min. Then, cells were incubated with 200 μl of primary antibody (1:200) overnight at 4 °C. The next day, cells were washed with 1× PBS followed by incubated with 200 μl of secondary anti-rabbit or anti-mouse antibodies (Alexa Fluor 488, #A11001 or Alexa Fluor 647, #A21235; 1:2000 dilution; Invitrogen Corporation) for 45 min in the dark. After incubation, cells were washed thrice with 1× PBS and mounted using DAPI on glass slides. Fluorescence images were captured using a super-resolution confocal microscope (Leica Stellaris 8 STED, Germany), using a 100x objective, and images were analyzed using LAS X image analysis software. Three separate sets of experiments were performed. Five images were taken from different arears of the coverslip for quantification.

### Evaluation of reactive oxygen species

Production of intracellular ROS was measured after DCFDA staining of live cells. In brief, RAW264.7 and primary BM cells (1 × 10^4^ cells/well) from mice were cultured on the coverslips for 6 days in differentiation media in the presence or absence of optimized concentration of EGCG. After 6 days of differentiation, coverslips were washed with HBSS and incubated with 100 µM of DCFDA solution for 30 min at 37 °C. Next, washed the coverslips thoroughly using 1× PBS, and thereafter, coverslips were mounted on the glass slide using DAPI. The fluorescence images were taken using a super-resolution confocal microscope (Leica Stellaris 8 STED, Germany), using a 100x objective, and images were analyzed using LAS X image analysis software. Three separate sets of experiments were performed. Five images were taken from different arears of the coverslip for quantification.

### Evaluation mitochondrial superoxide

MitoSOX red staining was performed for the detection of mitochondrial superoxide production. In brief, RAW264.7 and primary BM cells (1×10^4^ cells/well) isolated from mice were cultured on the coverslips using differentiation media with or without EGCG for 6 days. After differentiation, coverslips were washed with ice-cold 1× PBS and then incubated with mitoSOX red at a concentration of 2 µM for 30 min at 37 °C. After incubation and washing, coverslips were mounted on the glass slide using DAPI. The fluorescence images were captured using a super-resolution confocal microscope (Leica Stellaris 8 STED, Germany), with a 100x objective, and the images were analyzed using LAS X image analysis software. Three separate sets of experiments were performed. Five images were taken from different arears of the coverslip for quantification.

### Evaluation of mitochondrial membrane potential

JC-1 staining was performed to evaluate the mitochondrial membrane potentials. RAW264.7 and primary BM cells isolated from mice (1 × 10^4^ cells/well) were cultured on the coverslips for 6 days in OC differentiation media in the presence or absence of EGCG. The coverslips were washed with ice-cold 1× PBS and incubated with JC-1 dye for 20 min at 37 °C. Immediate after the incubation, coverslips were washed with 1× PBS and then mounted on the glass slide using DAPI. The fluorescence images were captured by a super-resolution confocal microscope (Leica Stellaris 8 STED, Germany), with a ×100 objective, and the images were analyzed using LAS X image analysis software. Three separate sets of experiments were performed for the staining. Five images were taken from different arears of the coverslip for quantification.

### ‘Seahorse Flux Analysis’ for evaluation of mitochondrial respiration and glycolysis

To assess the mitochondrial function during OC differentiation in the presence or absence of EGCG, we performed the cell MitoStress and glycolytic properties according to our previously described protocol by using an Agilent XFe^24^ flux analyzer [50, 51]. In brief, RAW264.7 (1 × 10^4^ cells/well) were cultured on a seahorse cell culture plate for 6 days in OC differentiation media with or without EGCG. On the day of the assay, assay media was prepared using Seahorse XF DMEM (Agilent, USA), supplemented with pyruvate, glutamine, and glucose. Removed the OC differentiation media and rinsed one time with assay media, and then added 500 µL of assay media in each well. Cells were incubated in a non-CO_2_ incubator at 37˚C for one hour before running the assay. Next, the assay was run, and oxygen consumption rate (OCR) and extracellular acidification rate (ECAR) using the standard and unmodified protocol and Seahorse Wave software 2.6.1 from Agilent was used for the analysis of the experimental data. Measurements were performed from 4 independent inductions for each time point. For determination of oxidative phosphorylation properties, the ATP synthase inhibitor oligomycin (1.5 μM), un-coupler carbonyl cyanide-4-(trifluoromethoxy) phenylhydrazone (FCCP, 1 μM), complex III inhibitor rotenone, and antimycin A (2 μM) was added to the wells. For determination of glycolytic properties, D-glucose (10 mM), oligomycin (1 μM), and glycolytic inhibitor 2-deoxy-D-glucose (2-DG, 50 mM) were also added accordingly. OCR and ECAR values were evaluated and calculated using the same software mentioned above.

### K/BxN serum-induced arthritis development in C57BL/6 mice

All murine experiments were conducted with prior Institutional Animal Care and Use Committee approval of TTUHSC, Lubbock, TX. To collect the arthritic serum for induction of inflammatory arthritis, K/BxN mice were generated by crossing KRN, TCR transgenic B6 mice (kind gift from Dr. Diane Mathis, Harvard Medical School, Boston, MA) with NOD mice (Jackson Laboratory) following the established protocol [52–54]. K/BxN serum was collected from 8-week-old arthritic K/BxN mice and pooled for each experiment. Each mouse (*n* = 6, C57BL/6 background 6–8 weeks old, female) was induced by intraperitoneal injection of 150 μl of K/BxN serum on days 0 and 2 following an earlier established protocol [55]. Equal amounts of PBS were injected into the control mice (*n* = 6). Mice were observed and ankle thickness was measured every day, and mice were finally sacrificed on day 8 following the first injection of K/BxN serum.

### Isolation of murine bone marrow and OC differentiation

After the termination of experiments, bone marrows (BM) were isolated from the hind limb of the mice. In brief, the mouse was euthanized and sterilized using 70% ethanol, followed by dissecting the mice and isolating the femur and tibia. HBSS was passed through the femur using 23-gauge needle to flush out the BM and collected into the 15 ml tube and centrifuged at 300×*g* for 5 min at 4 °C. Then BM cells were cultured overnight in αMEM complete media containing 20 ng/ml MCSF at 37°C incubator with 5% CO_2_. The next day, an equal number of non-adherent cells were cultured on coverslips in a well of a six-well plate for 6 days in a complete αMEM medium with 20 ng/ml MCSF combined with 50 ng/ml RANKL in the presence or absence of EGCG. Fresh media were used to replace them every 2 days of culture. After six days of differentiation DCFDA, mitoSOX, and JC-1 staining were performed according to the above-mentioned protocol.

### Molecular docking

The molecular interaction of EGCG with RANK was determined by using AutoDock 4.2 software. The three-dimensional (3D) structure of RANK and RANKL (PDB ID: 3ME2) was retrieved from RCSB Protein Data Bank (https://www.rcsb.org/) and the chemical structure of EGCG was downloaded from PubChem (https://pubchem.ncbi.nlm.nih.gov/). Prior to performing molecular docking, the undesirable water molecules and ligands associated with RANK were removed, polar hydrogen was added, and the energy was minimized using UCSF Chimera software. The structures of protein and ligand were further optimized using the AutoDock 4.2 tool and transformed into pdbqt format. A grid box (126, 126, and 126 Å) was defined on the macromolecule (RANK) and the grid size was screwed in *x, y*, and *z* directions to surround all the active amino acid residues of RANK usually interacted with RANKL. Lamarckian genetic algorithm (GA 4.2) was implemented to prepare for the docking file (.dpf) with all default settings. The prepared files were executed through AutoGrid 4.2 and AutoDock 4.2 scripts. The docked protein-ligand complex was analyzed and selected on the basis of high binding affinity, and lower inhibition constant. The binding Gibbs free energy (ΔG) and inhibition constant (Ki) are expressed as kcal/mol and micromolar (µM), respectively. Binding Gibbs free energy (ΔG) is calculated as a sum of energy terms of Vander Waals, electrostatic interaction, hydrogen bonding, desolvation effects, and internal ligand torsion-free energy. Discovery Studio Visualizer and PyMOL were used to get depth insight into the nature of interactions between RANK and EGCG.

### Statistical analysis

At least three separate sets of experiments were performed in a triplicate manner, and the results were shown as mean ± SEM. Statistical analyses were performed using Graph Pad Prism 9.0 for Windows (Graph Pad Software, San Diego, CA, USA). In the statistical analysis, a one-way ANOVA was used for Tukey’s multiple comparisons test. Unpaired two-tailed Student’s *t* tests were used for the comparison of means of data between two groups. *p* values <0.05 were considered significant.

## Supplementary information

Fig. S1.tiff

Fig. S2.tiff

Fig. S3.tiff

Fig. S4.tiff

Fig. S5.tiff

Fig. S6.tiff

Fig. S7.tiff

Fig. S8.tiff

Fig. S9.tiff

Fig. S10.tiff

Fig. S11.tiff

Fig. S12.tiff

Fig. S13.tiff

Original western blot R

AJ-CHECKLIST-filled

nr-reporting-sumary filled-HD signed

nr-editorial-policy-checklist-flat

nr-editorial-policy-checklist- HD signed

## Data Availability

The datasets used and/or analyzed during the current study will be available upon reasonable request.
