# Supplementary figures and images for: Epigallocatechin-3-gallate inhibits osteoclastic differentiation by modulating mitophagy and mitochondrial functions
Source: Cell Death Dis. 2022 Oct 28;13(10):908. doi: 10.1038/s41419-022-05343-1 (PMC9616829; doi:10.1038/s41419-022-05343-1)

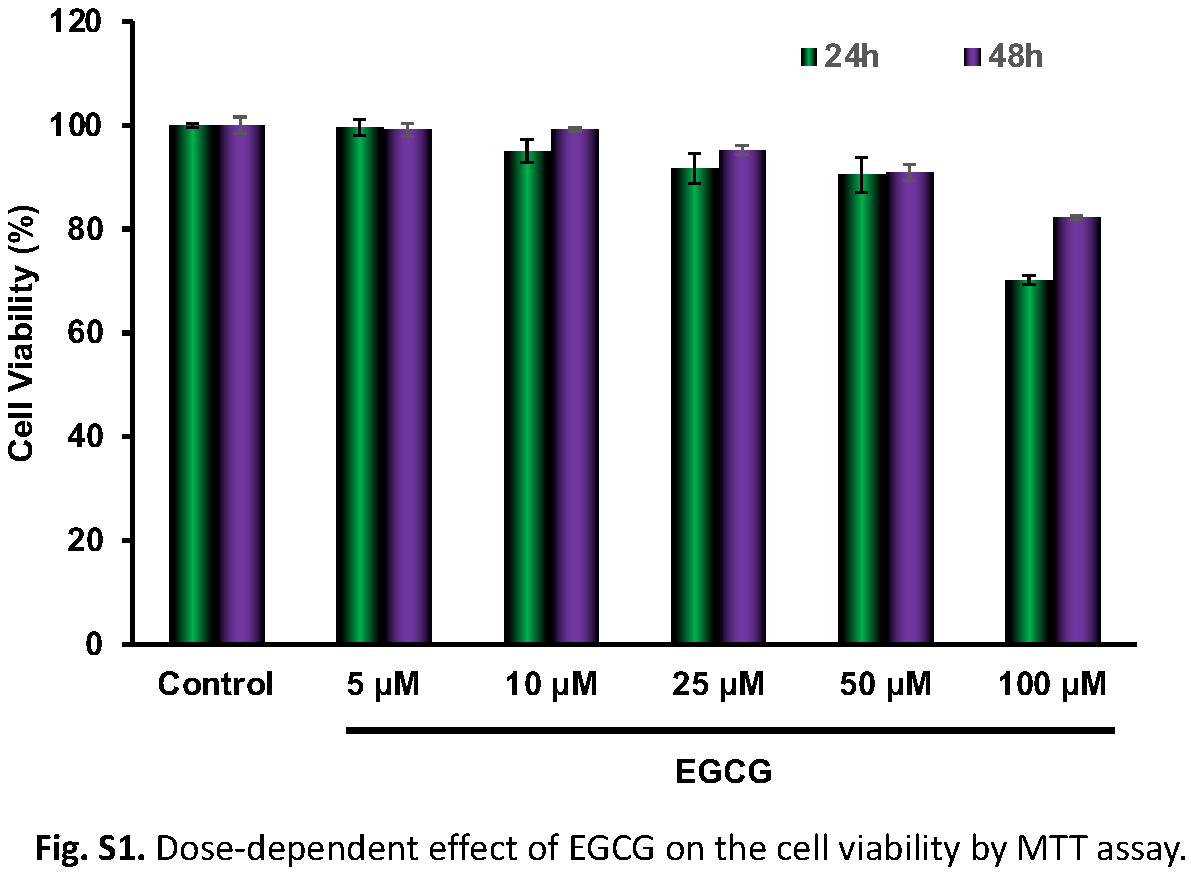

Supplement: Supplementary file 1 — Fig. S1.tiff [file 41419_2022_5343_MOESM1_ESM.tif]

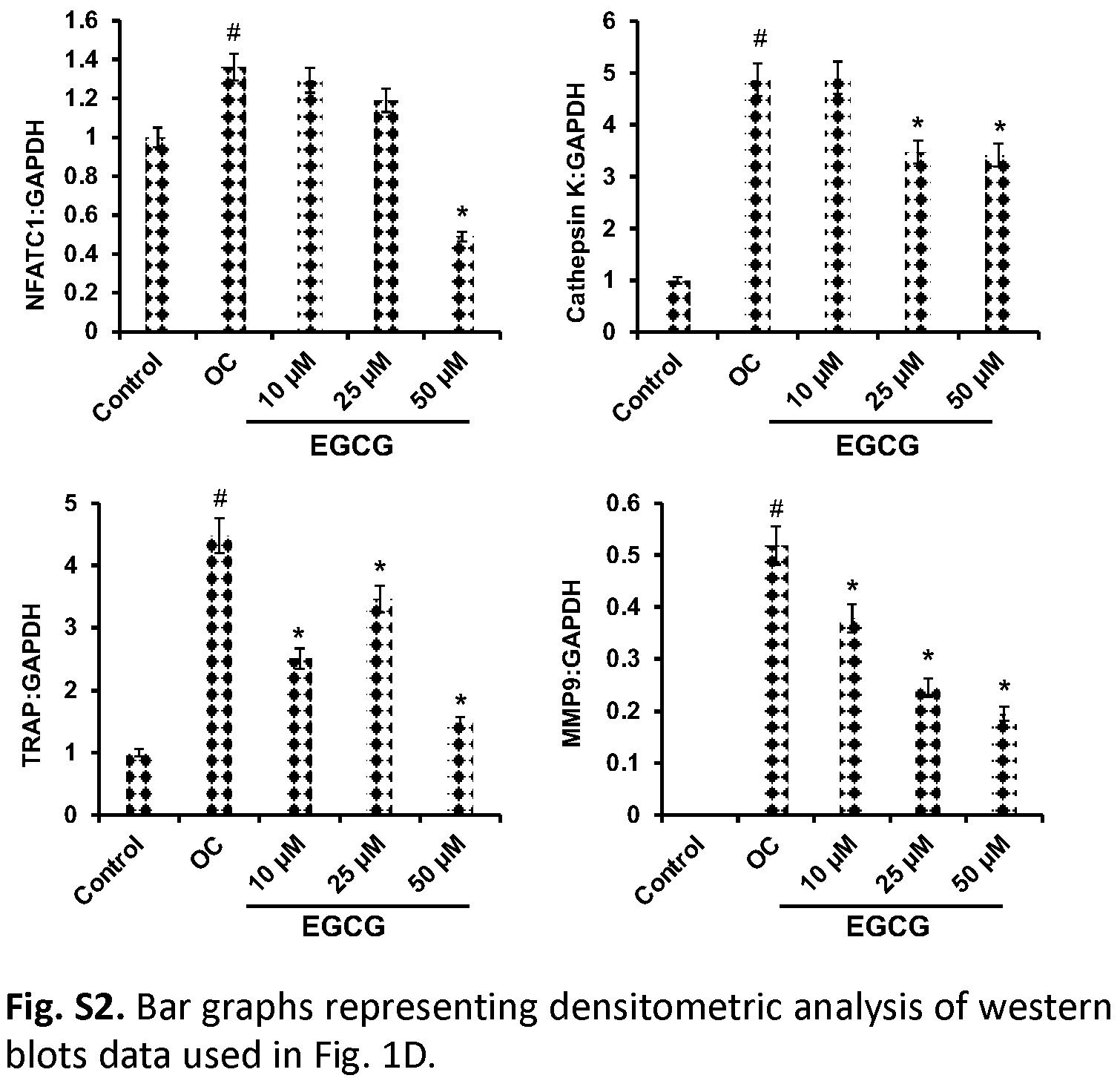

Supplement: Supplementary file 2 — Fig. S2.tiff [file 41419_2022_5343_MOESM2_ESM.tif]

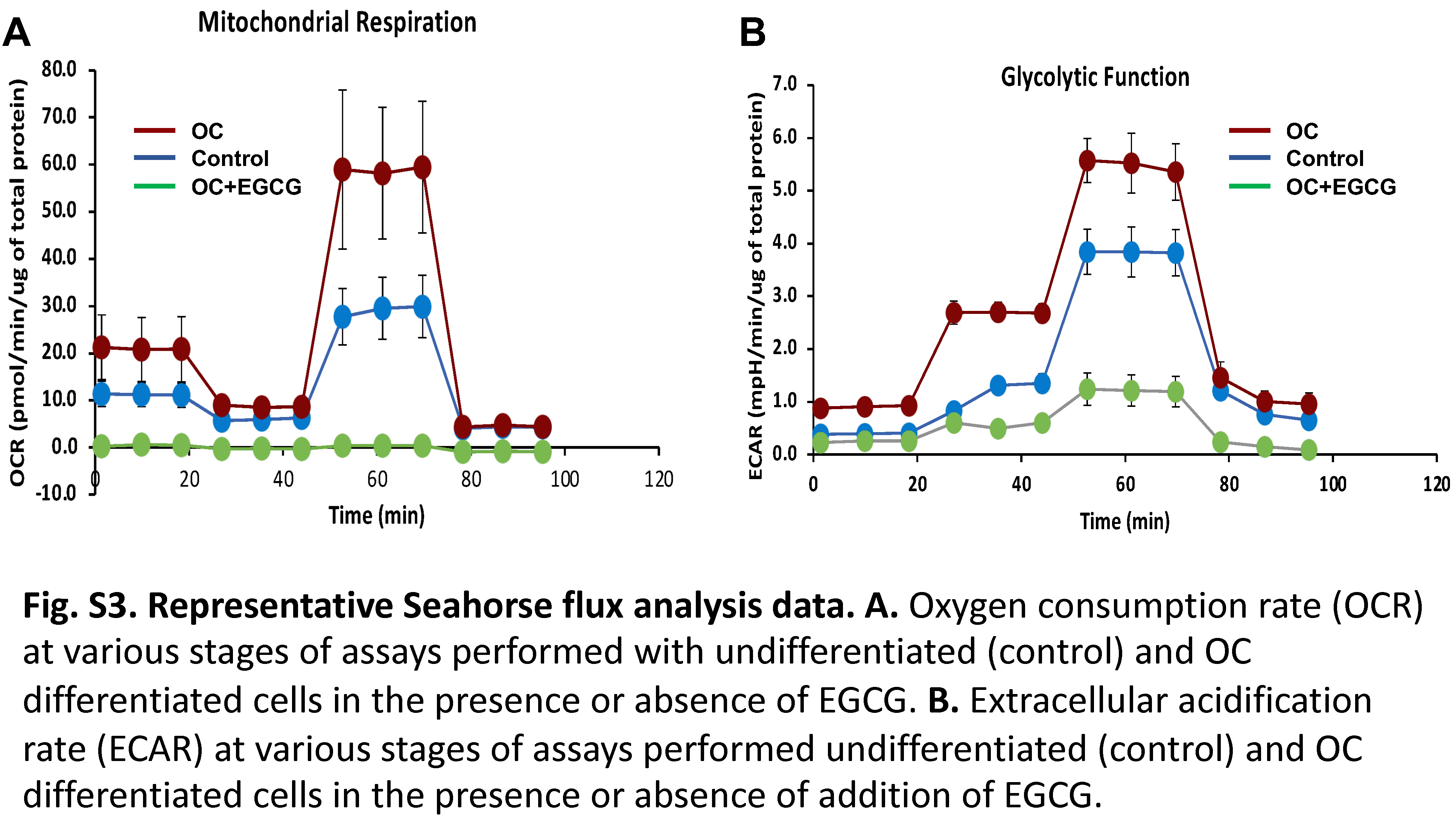

Supplement: Supplementary file 3 — Fig. S3.tiff [file 41419_2022_5343_MOESM3_ESM.tif]

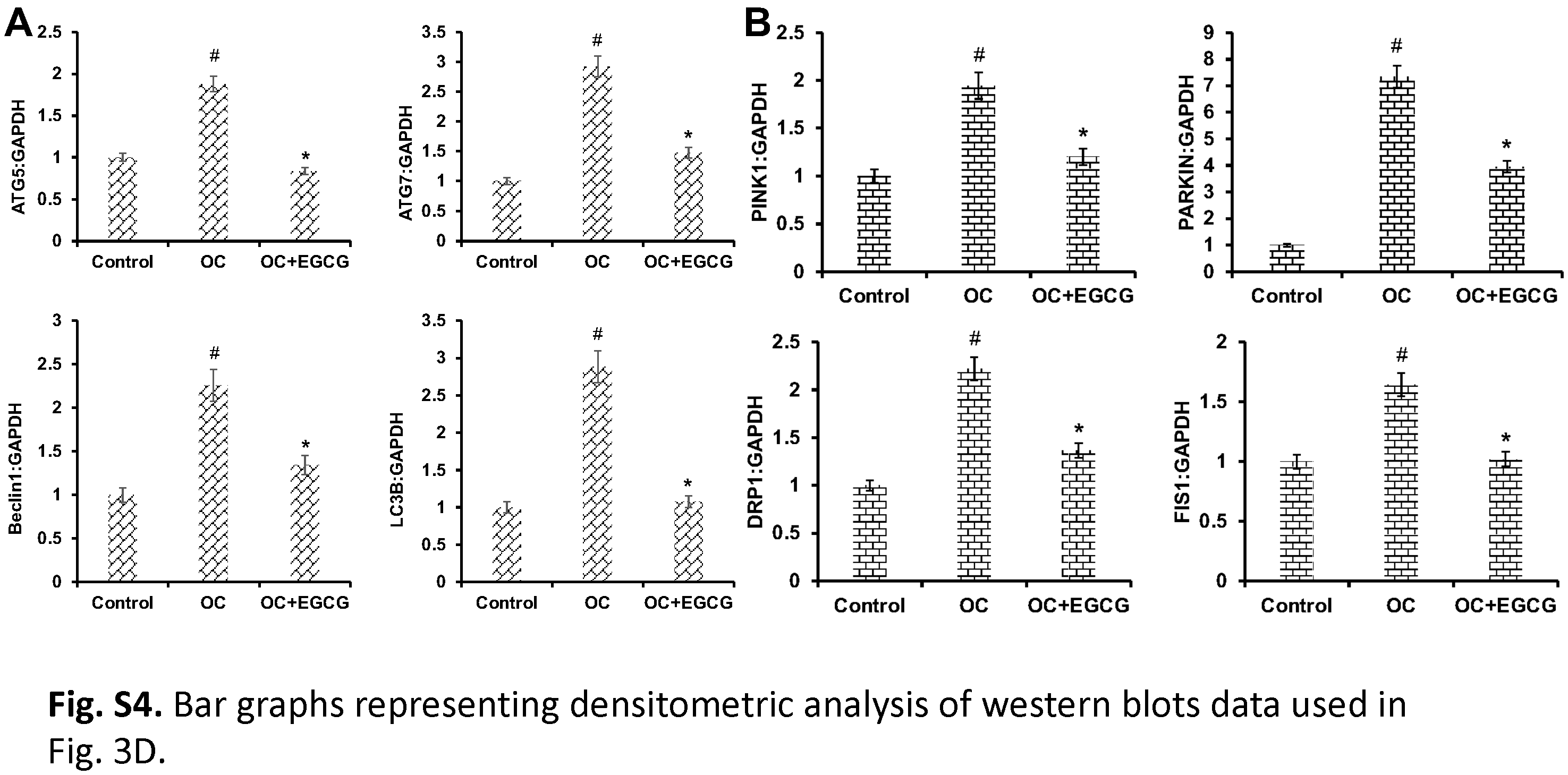

Supplement: Supplementary file 4 — Fig. S4.tiff [file 41419_2022_5343_MOESM4_ESM.tif]

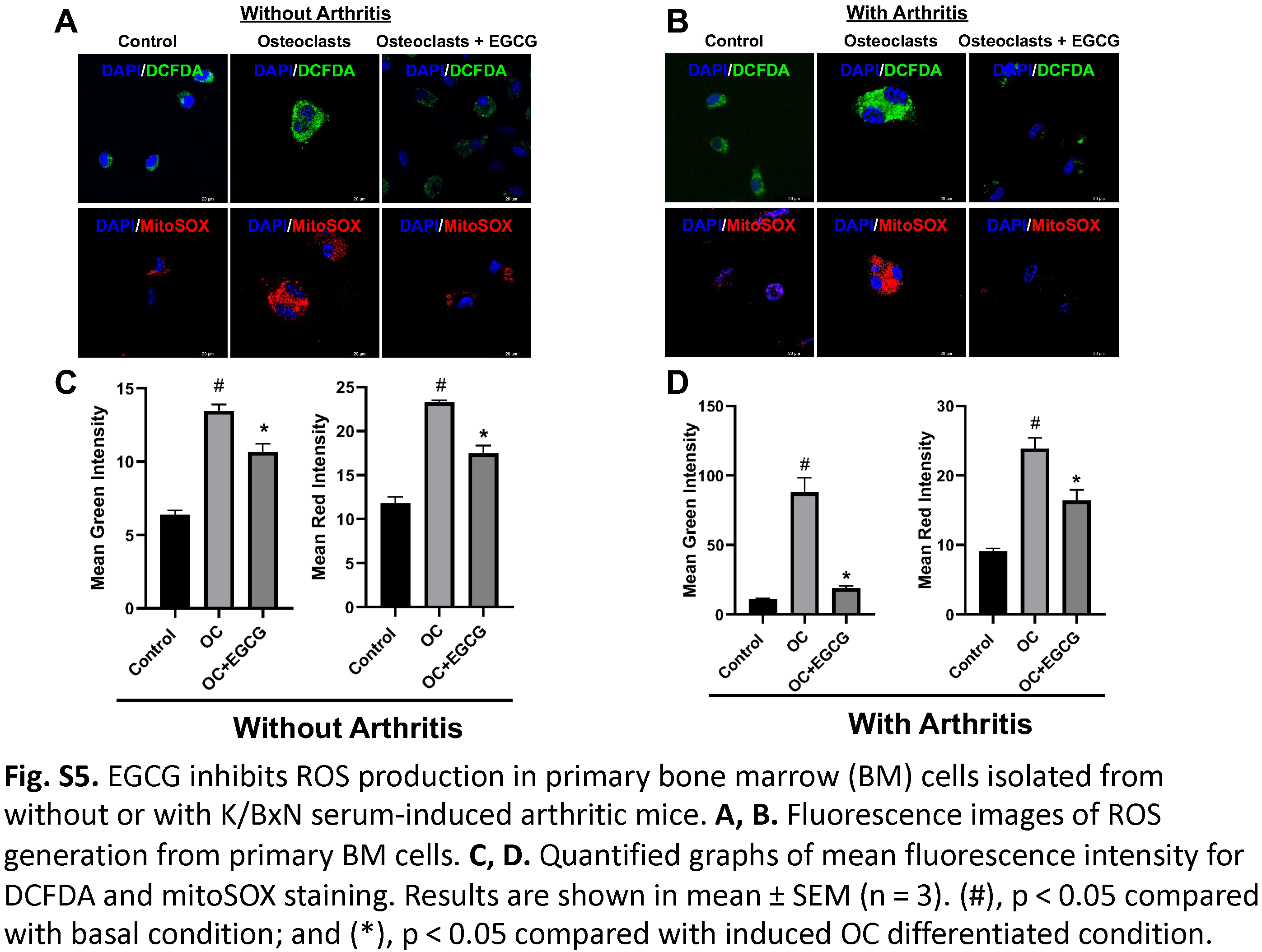

Supplement: Supplementary file 5 — Fig. S5.tiff [file 41419_2022_5343_MOESM5_ESM.tif]

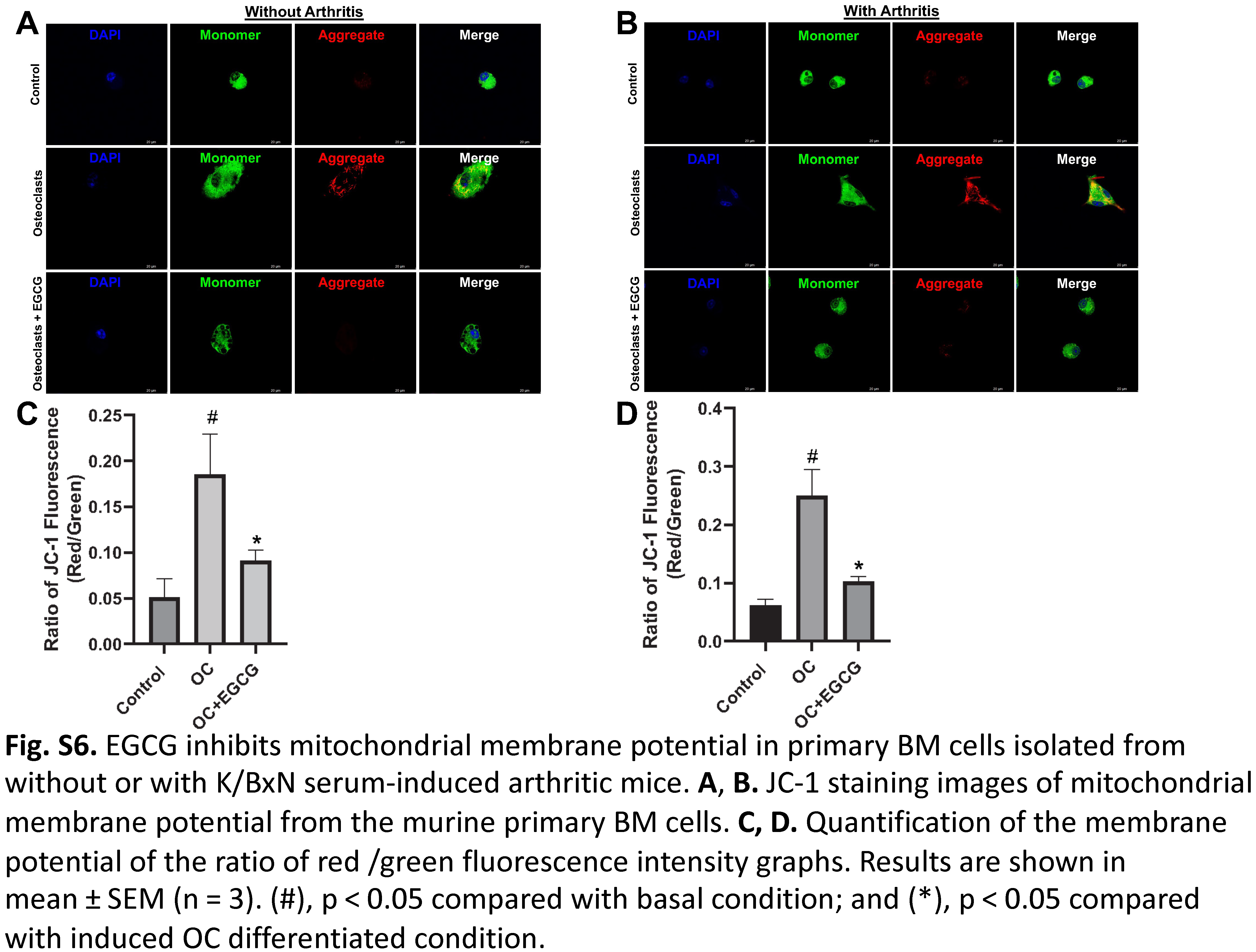

Supplement: Supplementary file 6 — Fig. S6.tiff [file 41419_2022_5343_MOESM6_ESM.tif]

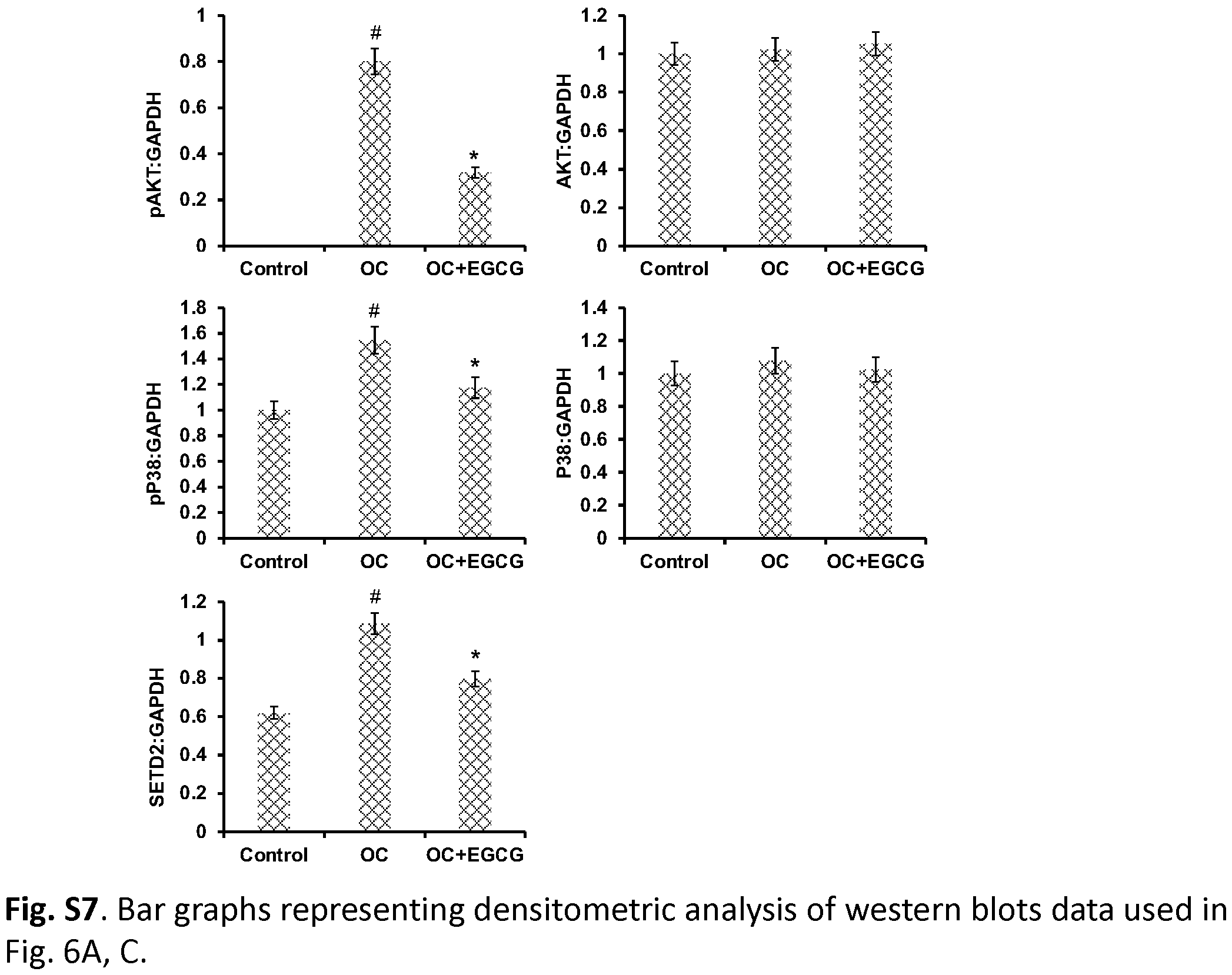

Supplement: Supplementary file 7 — Fig. S7.tiff [file 41419_2022_5343_MOESM7_ESM.tif]

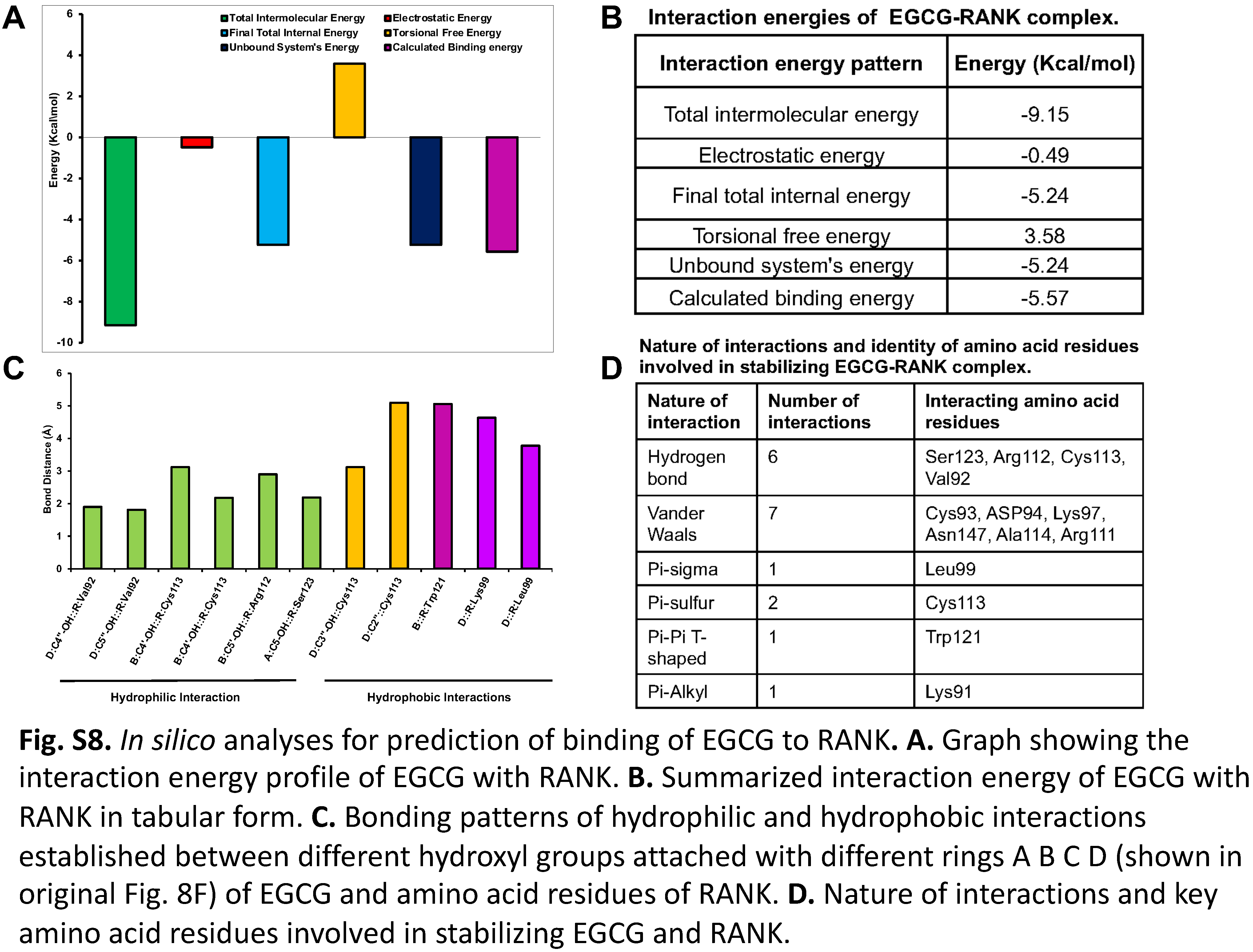

Supplement: Supplementary file 8 — Fig. S8.tiff [file 41419_2022_5343_MOESM8_ESM.tif]

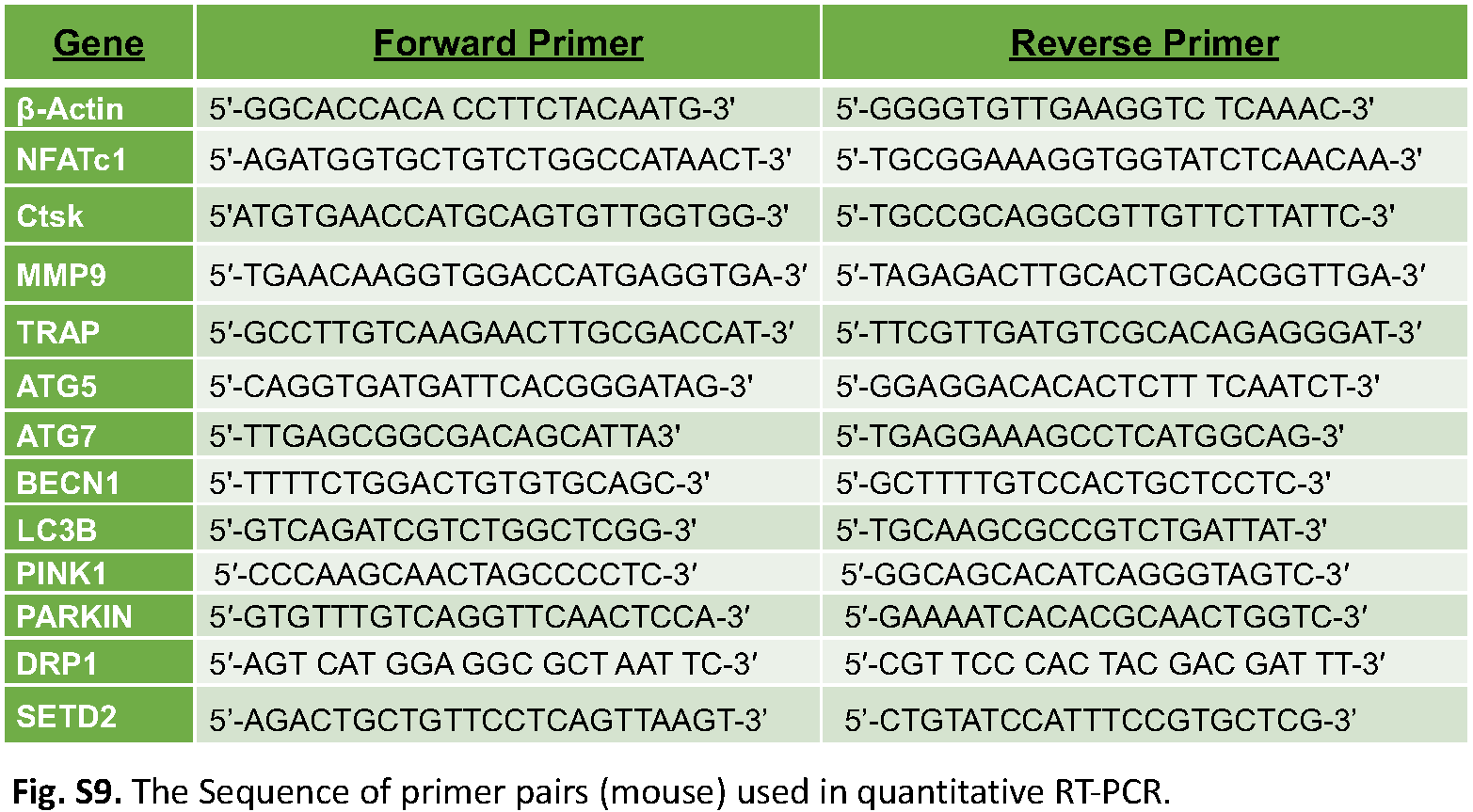

Supplement: Supplementary file 9 — Fig. S9.tiff [file 41419_2022_5343_MOESM9_ESM.tif]

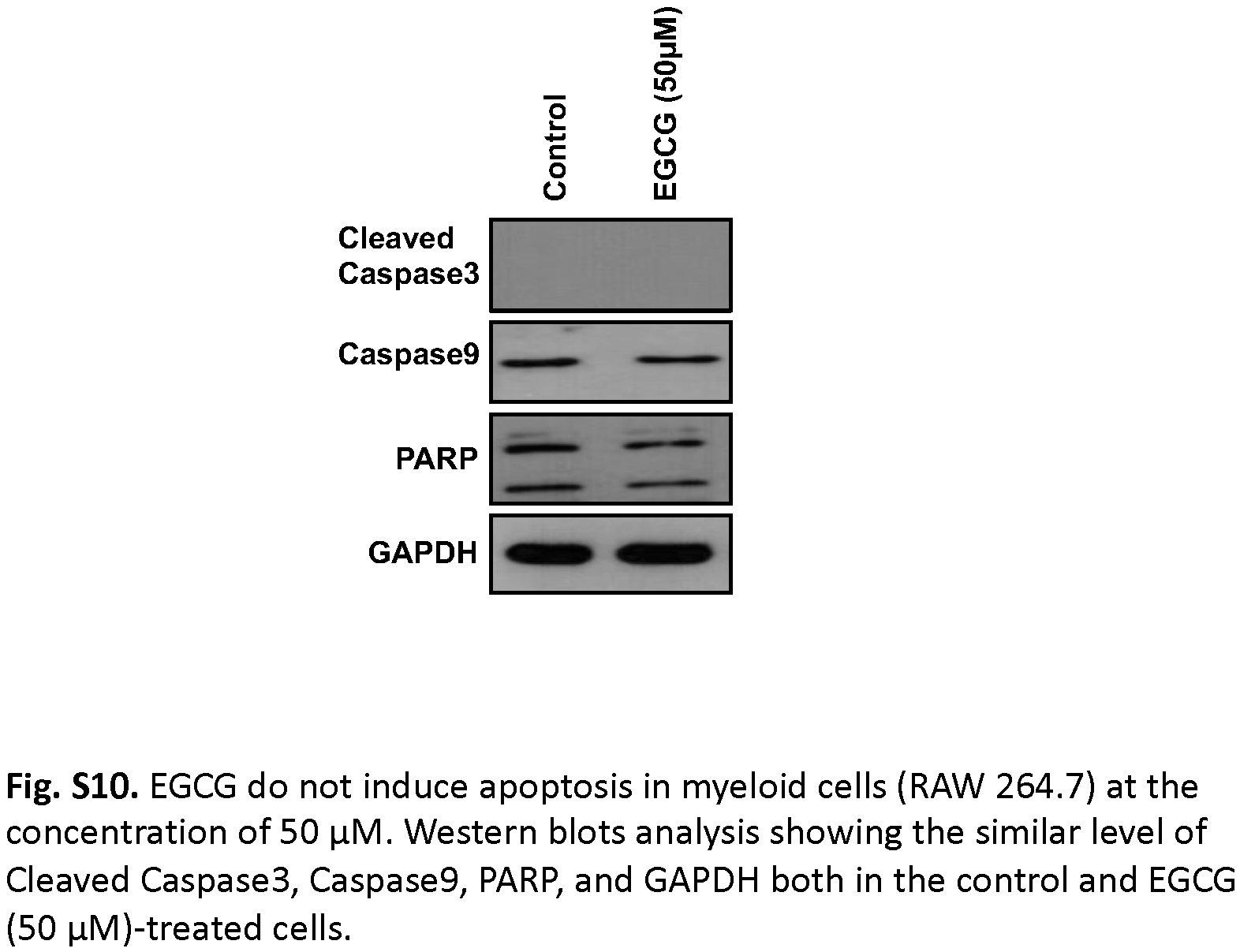

Supplement: Supplementary file 10 — Fig. S10.tiff [file 41419_2022_5343_MOESM10_ESM.tif]

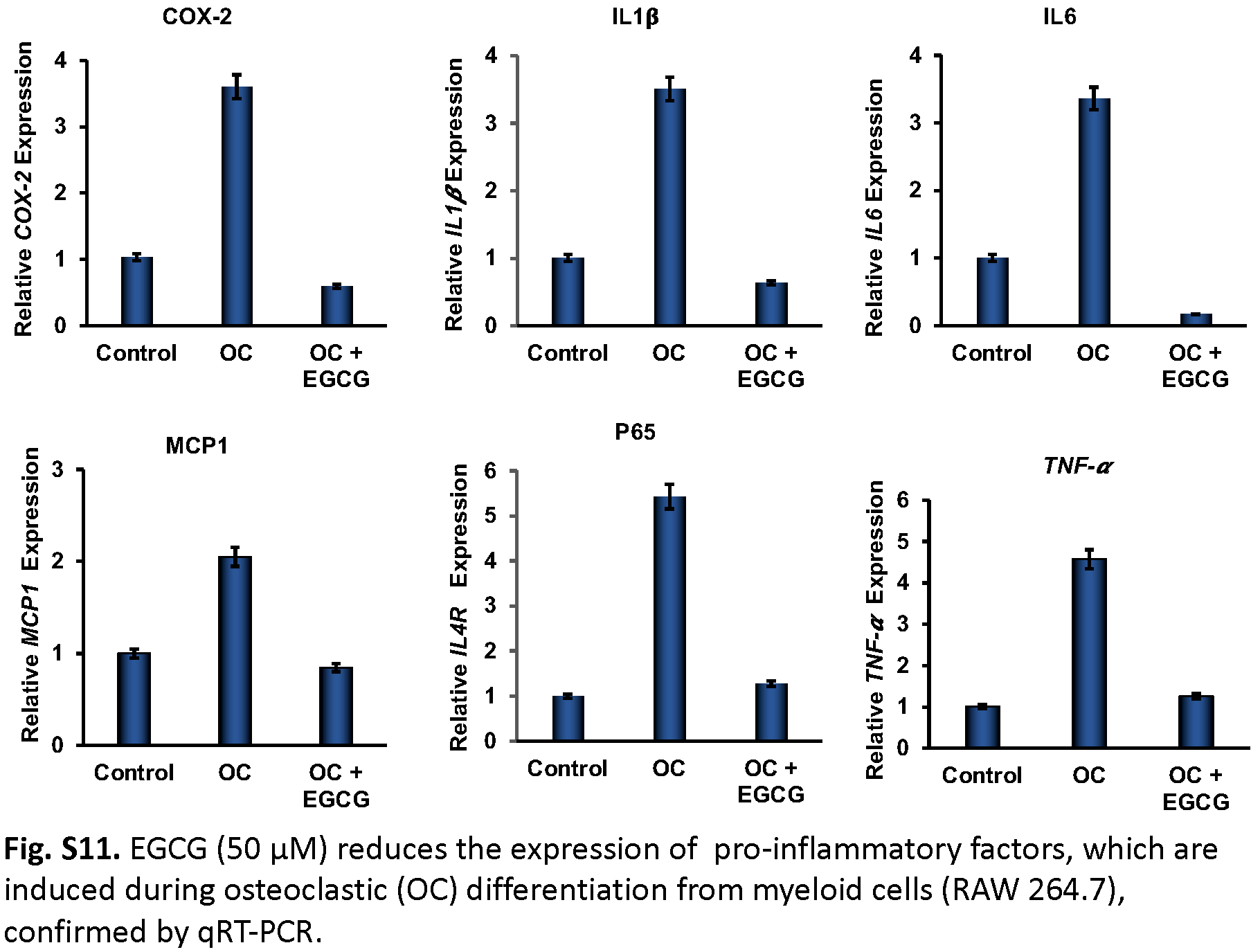

Supplement: Supplementary file 11 — Fig. S11.tiff [file 41419_2022_5343_MOESM11_ESM.tif]

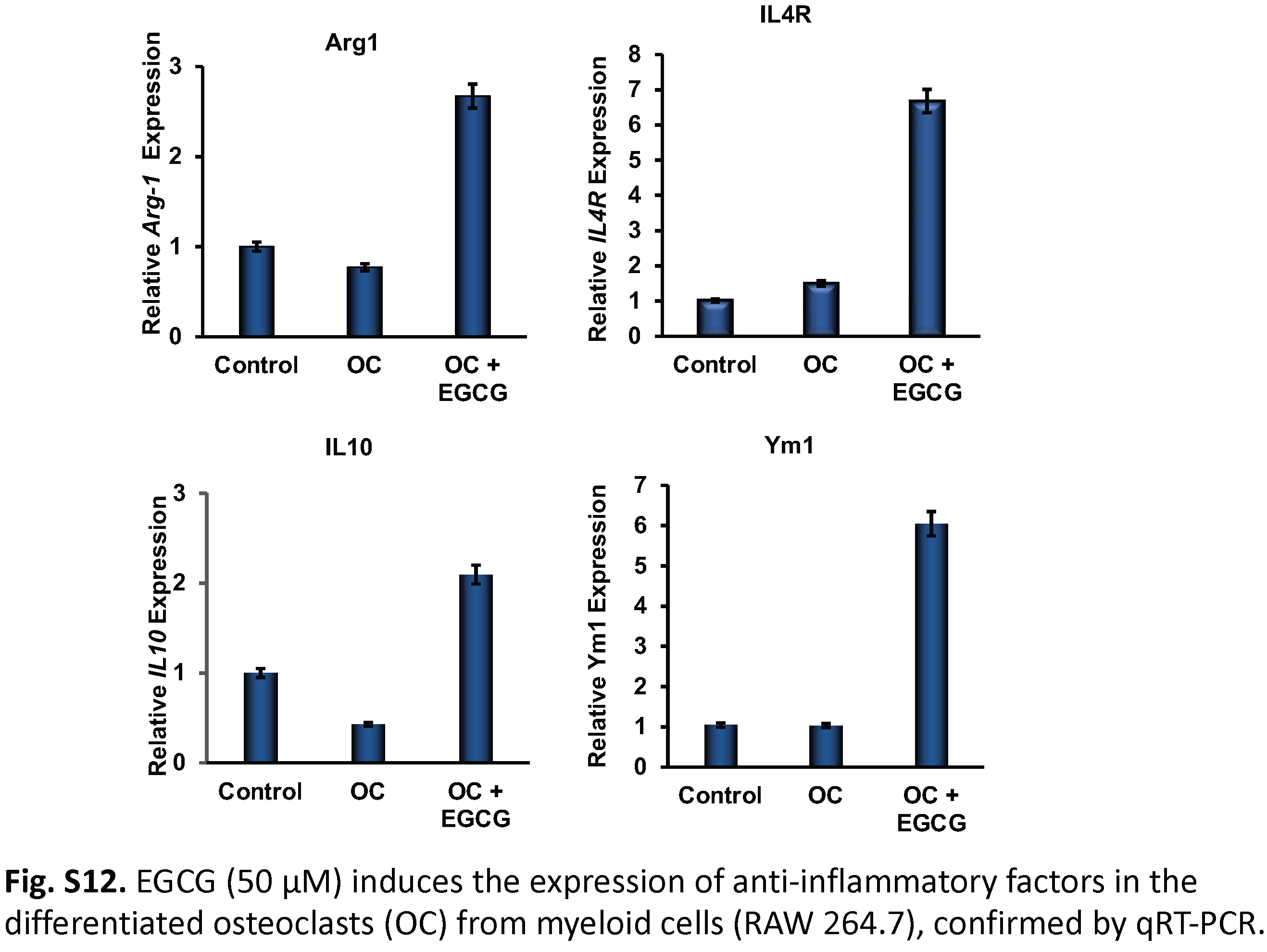

Supplement: Supplementary file 12 — Fig. S12.tiff [file 41419_2022_5343_MOESM12_ESM.tif]

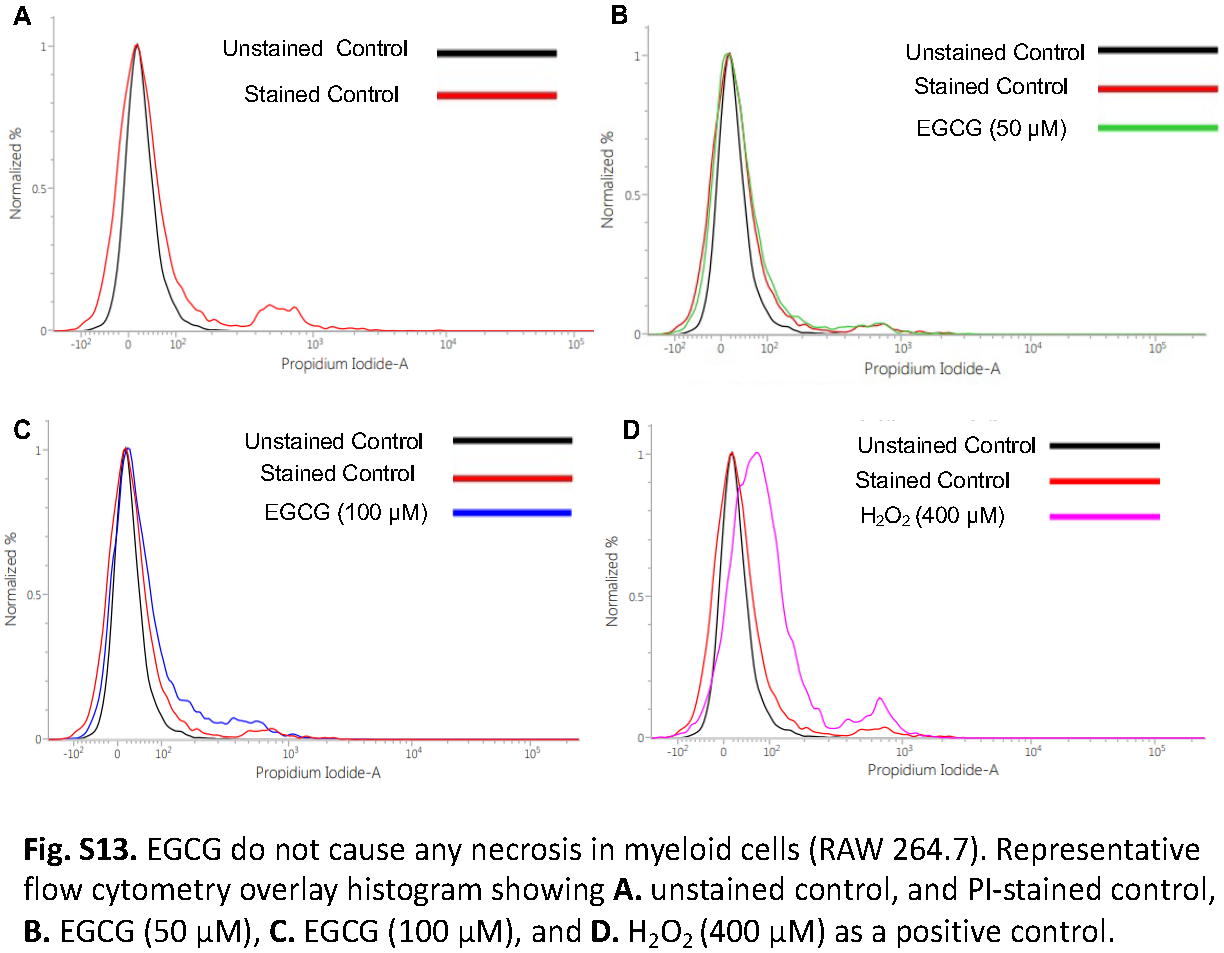

Supplement: Supplementary file 13 — Fig. S13.tiff [file 41419_2022_5343_MOESM13_ESM.tif]
