# Supplementary material for: Epigallocatechin-3-gallate inhibits osteoclastic differentiation by modulating mitophagy and mitochondrial functions
Source: Cell Death Dis. 2022 Oct 28;13(10):908. doi: 10.1038/s41419-022-05343-1 (PMC9616829; doi:10.1038/s41419-022-05343-1)
Supplement: Supplementary file 14 — Original western blot R [file 41419_2022_5343_MOESM14_ESM.pdf]

<u>Original western blots</u>

Epigallocatechin-3-gallate inhibits osteoclastic differentiation by modulating mitophagy and mitochondrial functions.

Jaganmay Sarkar, Manjusri Das, Md Sariful Islam Howlader, Prateeksha Prateeksha, Derek Barthels, and Hiranmoy Das*

Department of Pharmaceutical Sciences, Jerry H. Hodge School of Pharmacy, Texas Tech University Health Sciences Center, Amarillo, Texas, USA.

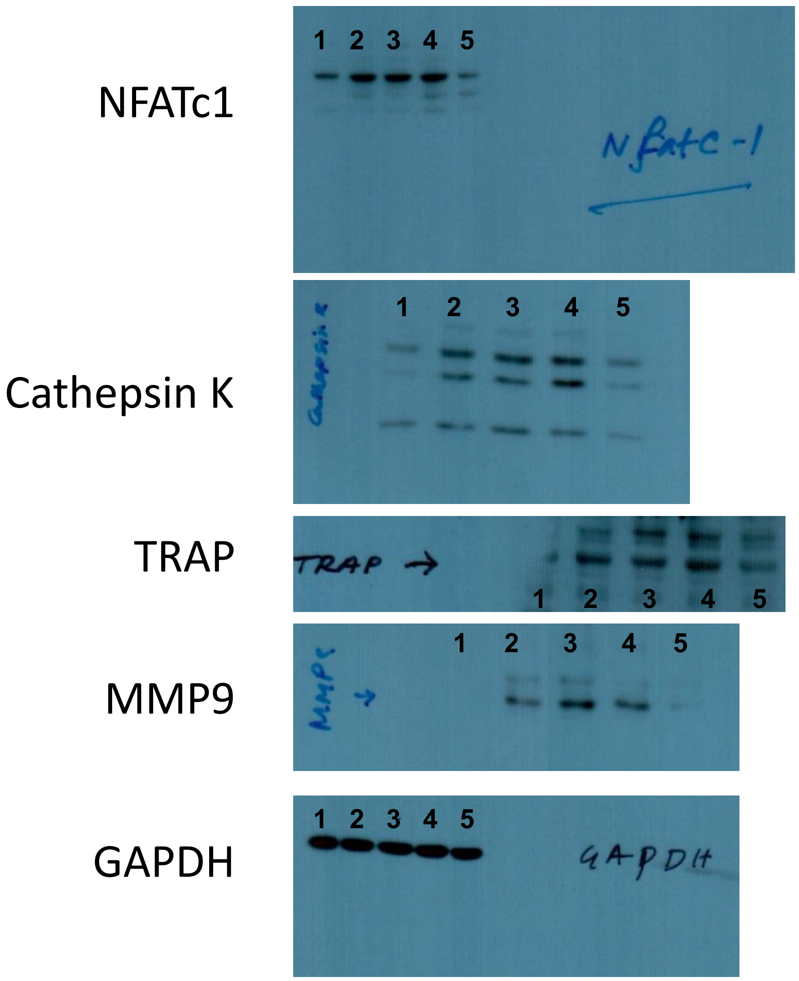

Lanes:

1 = Control
2 =OC
3 =OC + 10 μM EGCG
4 =OC + 25 μM EGCG
5 =OC + 50 μM EGCG

Fig. 1D

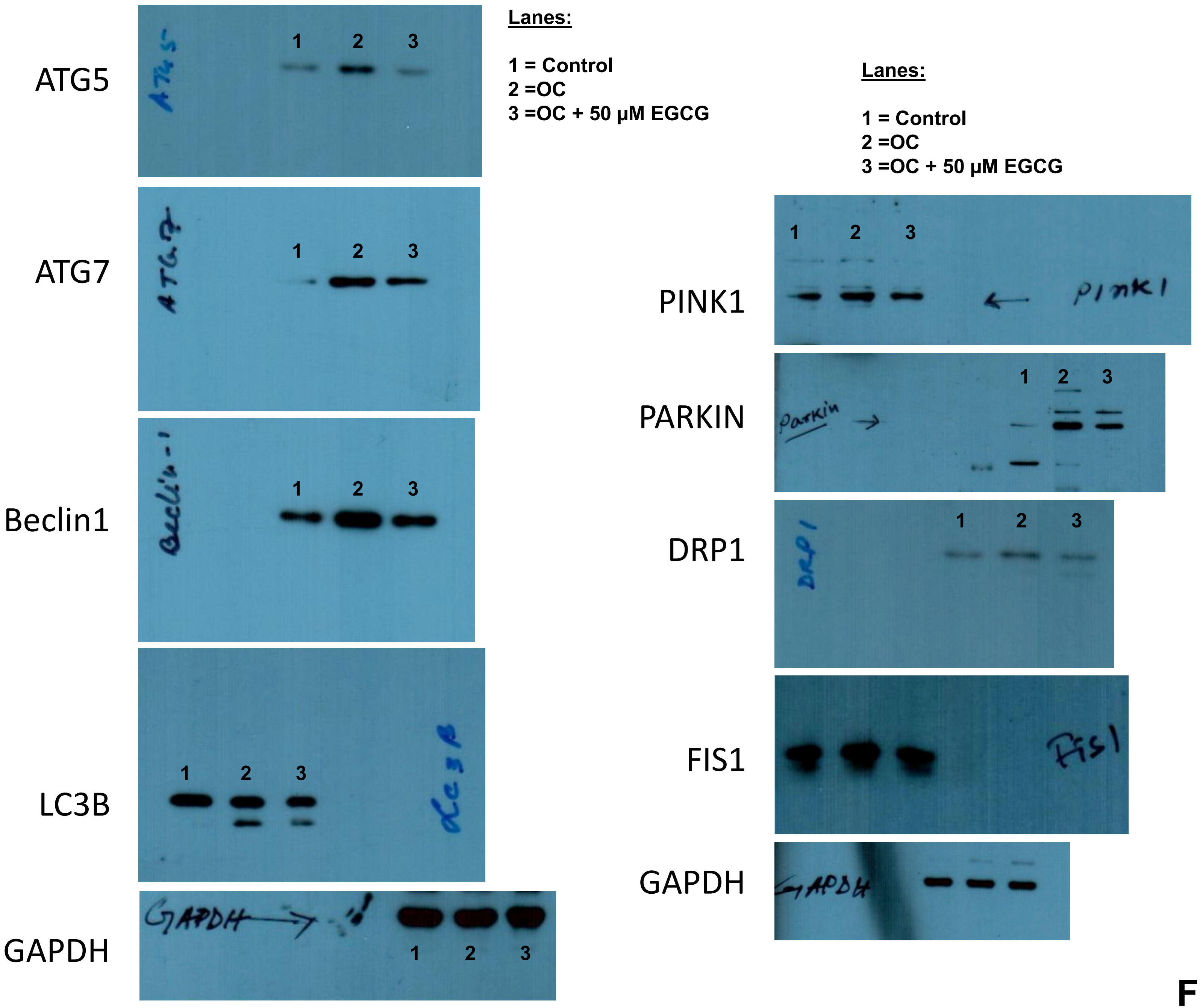
Lanes:
1 = Control
2 =OC
3 =OC + 50 µM EGCG
ATG5
ATG7
Beclin1
LC3B
GAPDH
Lanes:
1 = Control
2 =OC
3 =OC + 50 µM EGCG
PINK1
PARKIN
DRP1
FIS1
GAPDH

Fig. 3D

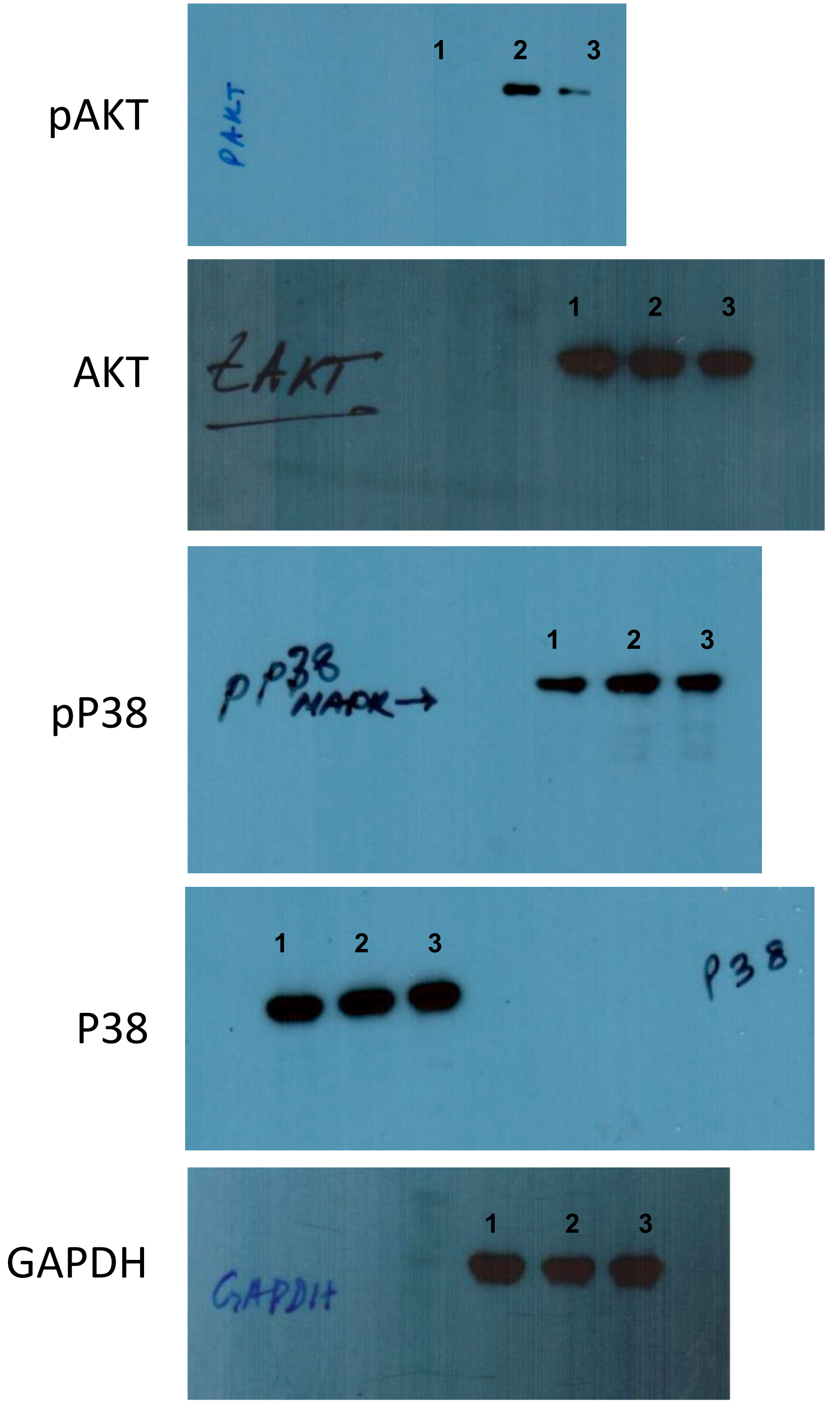

Lanes:

1 = Control
2 =OC
3 =OC + 50 μM EGCG

Fig. 6A

Lanes:

1 = Control
2 =OC
3 =OC + 50 μM EGCG

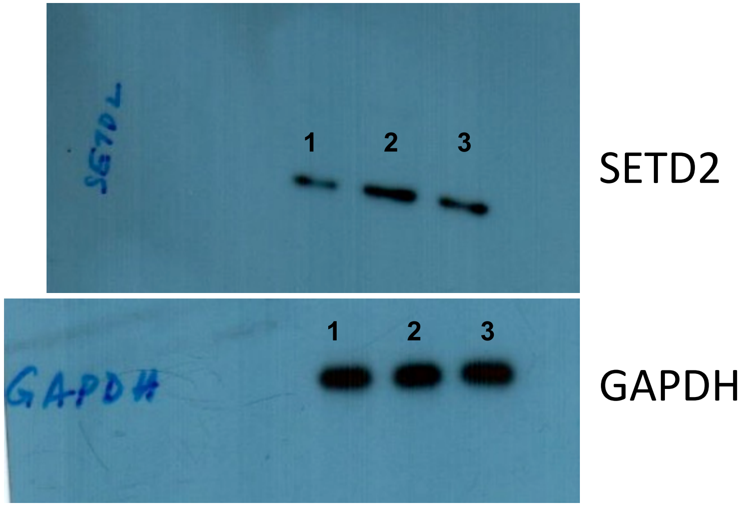

Fig. 6C

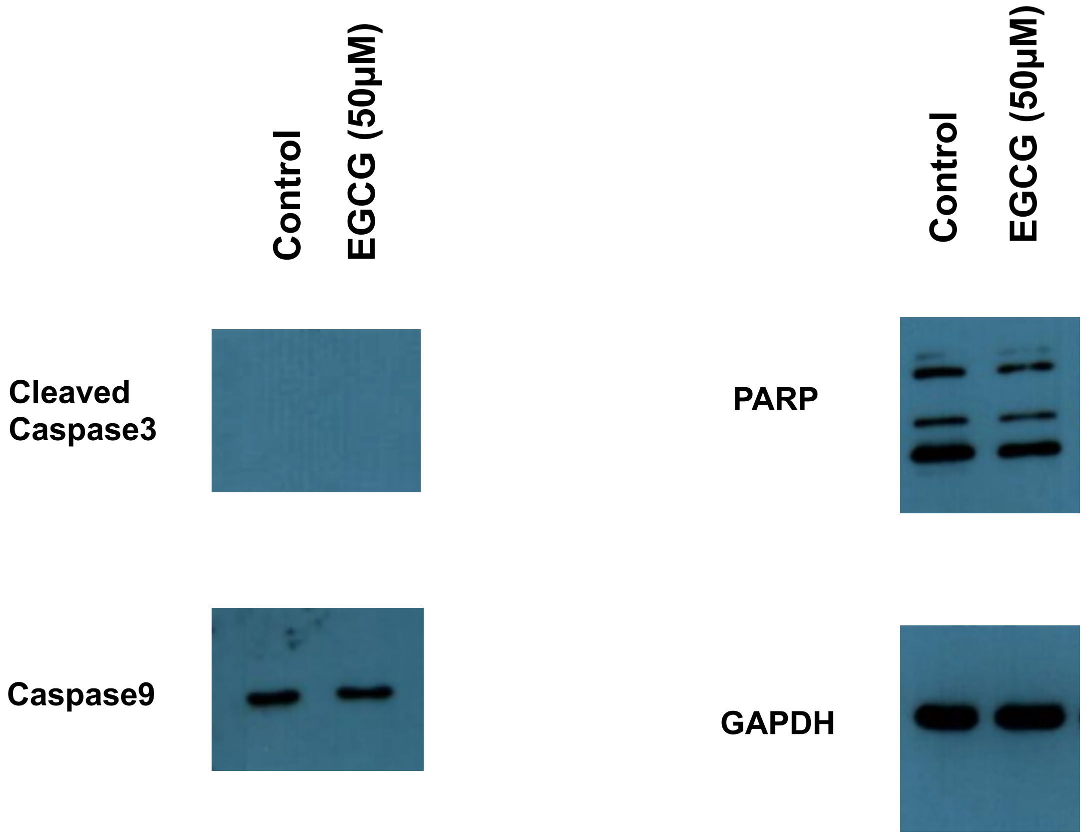

Control
EGCG (50μM)
Cleaved Caspase3
Caspase9
Control
EGCG (50μM)
PARP
GAPDH

Fig. S10
